# Contribution of autofluorescence from intracellular proteins in multiphoton fluorescence lifetime imaging

**DOI:** 10.1038/s41598-022-20857-6

**Published:** 2022-10-05

**Authors:** Monika Malak, Jeemol James, Julie Grantham, Marica B. Ericson

**Affiliations:** 1 Biomedical Photonics, Department of Chemistry and Molecular Biology, Faculty of Science, University of Gothenburg, Kemivägen 10, 412 96 Gothenburg, Sweden; 2 Department of Chemistry and Molecular Biology, Faculty of Science, University of Gothenburg, Medicinaregatan 9C, 413 90 Gothenburg, Sweden

**Keywords:** Optical imaging, Multiphoton microscopy, Cellular imaging, Intermediate filaments, Biophotonics, Biological fluorescence

## Abstract

Multiphoton fluorescence lifetime imaging microscopy (MPM-FLIM) is extensively proposed as a non-invasive optical method to study tissue metabolism. The approach is based on recording changes in the fluorescence lifetime attributed to metabolic co-enzymes, of which nicotinamide adenine dinucleotide (NADH) is of major importance. However, intrinsic tissue fluorescence is complex. Particularly when utilizing two-photon excitation, as conventionally employed in MPM. This increases the possibility for spectral crosstalk and incorrect assignment of the origin of the FLIM signal. Here we demonstrate that in keratinocytes, proteins such as keratin may interfere with the signal usually assigned to NADH in MPM-FLIM by contributing to the lifetime component at 1.5 ns. This is supported by a change in fluorescence lifetime distribution in KRT5- and KRT14-silenced cells. Altogether, our results suggest that the MPM-FLIM data originating from cellular autofluorescence is far more complex than previously suggested and that the contribution from other tissue constituents should not be neglected—changing the paradigm for data interpretation in this context.

## Introduction

Multiphoton laser scanning microscopy (MPM) is a technique operating in the near-infrared (NIR) regime^1^. MPM has gained substantial interest in biomedical research due to its ability to image biological tissues in a three-dimensional and noninvasive manner at a cellular resolution. The fluorescence signal in MPM is generated by non-linear optical excitation processes confined to a focal point, which then is scanned over the sample to create three-dimensional image sections. Process of two-photon excitation (2PE), among others, enabled by fs-pulsed laser light, is commonly utilized in MPM. Spectral separation and characterization of the fluorophores are primarily done with respect to their spectral emission, or fluorescence lifetime when combined with fluorescence lifetime imaging (FLIM)^2^.

Intrinsic cellular fluorescence (autofluorescence) is commonly considered a problem in the bioimaging community, as it interferes with the fluorescence from exogenous staining probes^3–5^; however, in the case of live tissue investigations, the intrinsic fluorescence becomes beneficial as it enables label-free noninvasive imaging^6,7^. In the skin, the main cell type contributing to tissue homeostasis and metabolism are keratinocytes, the building blocks of the epidermis. The epithelial layer, being comprised of differentiating keratinocytes, provides a mechanical and chemical barrier from the outside environment^8^, while the fibroblasts, the main cell type present in the dermis, generate the underlying supportive connective tissue. Paracrine interactions between those cell types have been previously described, where the cells stimulate each other through growth factor signaling, and coordinate wound healing processes^9–11^. When using MPM for skin studies based on 2PE of intrinsic fluorescence, the primary signal generators are believed to be nicotinamide adenine dinucleotide (NADH) and flavin adenine dinucleotide (FAD). Additionally, other components such as keratin, collagen, elastin, or melanin have been reported to contribute to the signal^12^.

Proteins have been shown to exhibit intrinsic fluorescence due to the presence of aromatic amino acids^13^, where tryptophan has been presented as the main contributor to the protein fluorescence^14^ and the potential source of the energy transfer^15^, suggesting that metabolic investigations of cellular and tissue environments may be more challenging than anticipated. Several reports have shown that the metabolic changes related to the cellular redox ratio^16^ based on NADH and FAD can be monitored with MPM-FLIM in a number of models, e.g., in precancerous epithelia^17^ or stem cells undergoing adipogenic differentiation^18^. However, when translating the findings in the context of the tissue, the complexity of the environment increases, potentially leading to spectral crosstalk, and increasing the risk of erroneous signal assignment, if one is to assume that NADH and FAD are the only fluorophores contributing to the intrinsic fluorescence. In keratinocytes, keratins are the intermediate filament proteins providing the cell with mechanical strength and resilience^19^, and have been reported to display intrinsic fluorescence in MPM of skin^20^ and hair^21^. However, little is known on the origin of keratin fluorescence or the effect of keratin intermediate filaments on the MPM-FLIM imaging. It has been speculated that the fluorescence originates from the energy transfer, through the “diffusion of photochemical products, exciton migration, or the process involved in sensitized fluorescence”^22^. Furthermore, it is likely that the non-linear optical processes taking place while using MPM are less specific, leading to multiple fluorescence-based processes in the keratin filaments, which are not widely known or understood. Therefore, before commencing MPM-FLIM metabolic studies on skin, the fluorophore composition and subsequent spectral and lifetime crosstalk needs to be investigated on the cellular level.

The spectral characteristics of the intrinsic fluorophores NADH, FAD and keratin reported in the literature, are summarized in Table 1. According to the published data, NADH and FAD can be distinguished by their emission spectra, although spectral crosstalk with keratin can be anticipated. In addition, 2PE of intrinsic fluorophores is complex. Much about the fluorophore interaction and behavior in the tissue is still unknown, especially in relation to how the spectroscopic information can be correlated with biochemical composition. Thus, this potential crosstalk needs to be carefully investigated in order to interpret spectral information from keratinocytes.

**Table 1:** Spectral characteristics and lifetime values of intrinsic cellular fluorophores expected in keratinocytes.

Fluorophore	Specimen	Excitation wavelength (nm)	Emission wavelength (nm)
NADH	NADH solution^23^ NADH solution, skin biopsy^24^ Isolated NADH^25^ NADH solutions^26^ Endothelial cells^27^ NADH solution, cells^28^ NADH solution, cells^29^	355 (1P)^23^ 355–395 (1P), 710–790 (2P)^24^ 366 (1P)^25^ 335–340 (1P)^26^ 375 (1P)^27^ 720–890 (2P)^28^ 740 (2P)^29^	470 (peak)^23^ 460 (peak)^24^ 450–470^25^ 430–470^26^ 440–480^27^ 425–475^28^ 440–480^29^
FAD	FAD solution^30^ FAD solution, skin biopsy^24^ Isolated FAD^25^ FAD solution, cells^28^	355 (1P)^30^ 415 (1P), 830 (2P)^24^ 405–500 (1P)^25^ 720–890 (2P)^28^	– 530 (peak)^24^ 520–560^25^ 520–600^28^
Keratin	Keratin solution, skin biopsy^24^ Purified keratin solution, skin biopsy^20^ skin biopsy, human hair^21^	355–415, 710–830^24^ 725–800^20^ 750^21^	400–520^24^ 425–500^20^ –

A number of reports on the fluorescence lifetime characteristics of NADH and FAD are available, whilst the data for keratin is limited. According to the literature, the fluorescence lifetime of NADH and FAD varies depending on whether the molecule occurs in its free or bound form (i.e., bound to enzymes and/or proteins). This reported difference in lifetimes is the foundation of a number of methodologies, e.g., the phasor approach, to utilize FLIM for non-invasive studies of cell metabolism^31^ and lipid peroxidation^32^. The lifetime values for NADH vary between 0.2 and 1.2 ns for its free form, while the bound NADH is reported to be in the range of 1.0 ns to 6.5 ns^23,26–28^. The lifetime of FAD has been previously reported to be in the range of 2.3 to 3.6 ns for the free form, and 0.3 to 0.7 ns in its bound form^30,33–35^. As for keratin, the fluorescence lifetime has been reported to be around 1.4 ns^21^. The reported lifetime range for NADH and FAD shows a large variability between the studies and it is particularly challenging to acquire spectral data from the bound only species^29^. Together, these factors may present a high risk of incorrect assignment of the origin of the signal if other cellular fluorophores are present.

We have previously presented autofluorescence MPM as a tool for label-free investigation of in vitro epidermal differentiation based on tissue morphology^36^. In vitro epidermis models cultured in varying calcium concentrations exhibited substantial morphological and structural differences. To allow for suitable noninvasive monitoring of organogenesis and tissue regeneration on a metabolic level, additional spectral and biochemical information is required. Before the approach can be adapted to a complex 3D environment, the spectral and lifetime crosstalk needs to be investigated at the cellular level. Therefore, the aim of this study was to characterize the intrinsic cellular fluorescence in keratinocytes using 2PE and MPM-FLIM. Of particular interest was to define the contribution of keratin to the MPM-FLIM readout in the cells. Spectral and lifetime characteristics of keratinocytes were compared with pure fluorophores in solution and cell cultures of fibroblasts. Ultimately, the impact of keratin on the MPM-FLIM signal was investigated by siRNA silencing of KRT5 and KRT14 genes.

## Results and discussion

### Spectral and FLIM separation of intrinsic fluorophores

**Figure 1:**
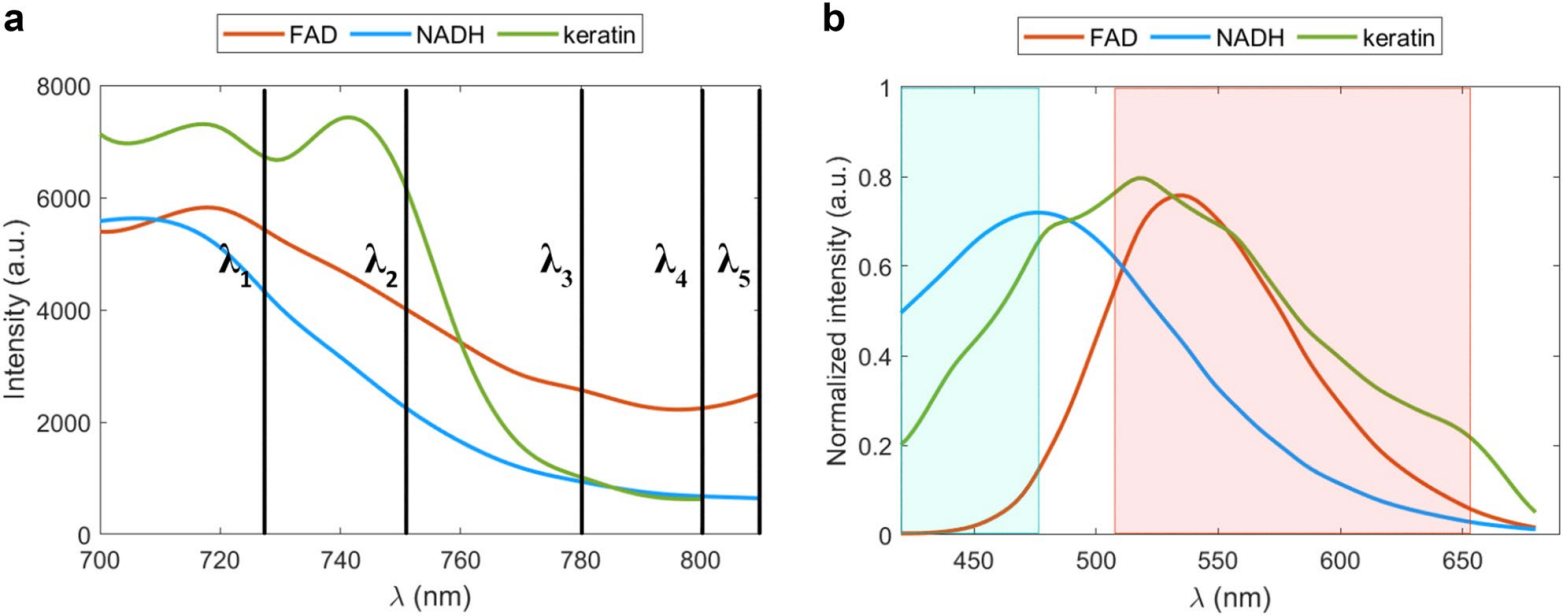
Two-photon excitation and emission spectra of the fluorophores in solution. (**a**) Excitation spectra of FAD (orange), NADH (blue), and keratin (green) in the excitation range of 700–820 nm. The excitation wavelength chosen for the study: λ_1_—725 nm, λ_2_—750 nm, λ_3_—780 nm, λ_4_—800 nm, and λ_5_—810 nm. (**b**) Normalized emission spectra of FAD (orange) and NADH (blue) excited at 720 nm, and keratin (green) excited at 700 nm. The range of the filters used is indicated by the blue- and red-shaded regions.

Two-photon excitation and emission spectra were acquired from the fluorophores in solution to confirm the spectral properties of NADH, FAD, and keratin. All three fluorophores exhibit 2PE in the range of 700 to 810 nm (Fig. 1a). Notably, keratin exhibits a higher probability for 2PE over FAD and NADH when increasing the excitation wavelength in the range of 700 to 760 nm. The emission spectra of all three fluorophores are broad and overlapping (Fig. 1b). The emission spectrum of NADH peaks at around 470 nm, while FAD shows a maximum emission at around 530 nm, confirming that these two fluorophores can potentially be spectrally distinguished (in agreement with Table 1); however, the spectral emission of keratin overlaps with emission from both FAD and NADH, potentially leading to spectral crosstalk. It is expected that the respective contribution of the fluorophores will fluctuate depending on their intracellular distribution and concentration in the cellular environment. In these experiments, the concentration of keratin was 11 mg protein/ml, while the overall keratin concentration in basal keratinocytes has been reported to be ~ 40 mg/ml ^37^, suggesting that the potential contribution from intrinsic keratin fluorescence can be substantial when performing MPM on keratinocytes and epidermal tissue.

Two spectral regions, blue (445/60 nm) and red (580/150 nm), were introduced to separate the spectral emission of the fluorophores in MPM-FLIM imaging, as indicated in Fig. 1b. According to the spectral measurements, the blue channel should primarily collect the emission from NADH and keratin, while in the red channel the emission from all three fluorophores is expected. The different excitation wavelengths used for the MPM-FLIM measurements are shown in Fig. 1a as λ_1-5_, corresponding to: 725 nm, 750 nm, 780 nm, 800 nm, and 810 nm.

**Figure 2:**
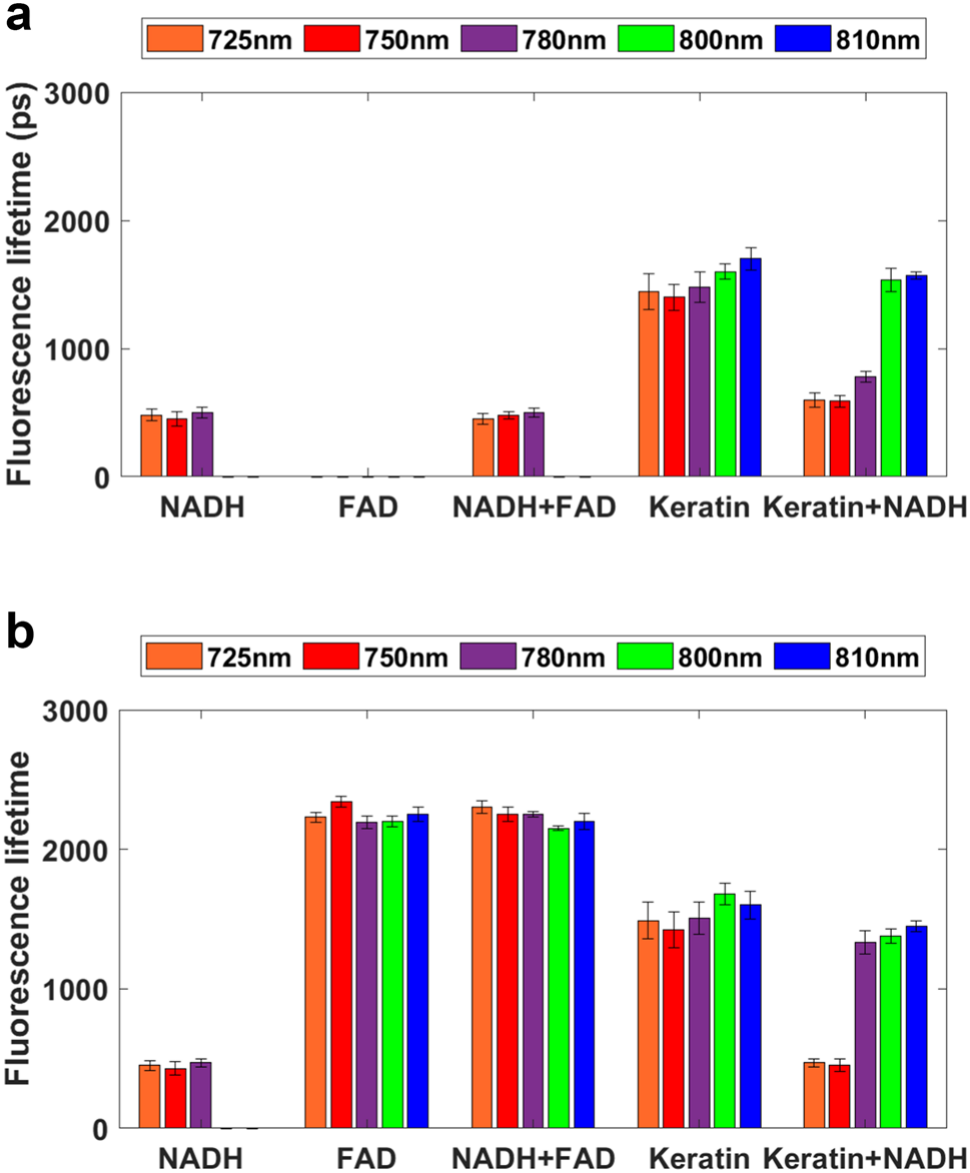
Fluorescence lifetime values from the fluorophores in solution. The average lifetime values are presented for the following solutions: NADH (150 µM), FAD (100 µM), NADH and FAD mixture (150 µM and 100 µM, respectively), keratin (180 µM), and keratin and NADH mixture (140 µM and 150 µM, respectively), collected in (**a**) the blue channel (445/60 nm), and (**b**) the red channel (580/150 nm) at the excitation wavelengths of 725 nm,750 nm,780 nm, 800 nm, and 810 nm. Single exponential decay was employed for one-component solutions, while biexponential decay model was used for the two-component solutions, presented as amplitude weighted average lifetime.

The average fluorescence lifetime values of NADH, FAD, and keratin in solution, together with two-component mixtures (NADH/FAD and NADH/keratin), were acquired using MPM-FLIM in the blue (445/65 nm; Fig. 2a) and red (580/150 nm; Fig. 2b) spectral channels. Detailed fluorescence lifetime distribution histograms from both channels at all excitation wavelengths are presented in the Supplementary material (Figs. S1 and S2). As expected, NADH exhibited a fluorescence lifetime value at approximately 0.5 ns in both channels in the excitation range 725–780 nm, consistent with values previously reported for free NADH^38^. Increasing the concentration of NADH from 150 to 5 mM expanded the signal detection in the studied excitation range (Fig. S1), indicating that the signal detection is concentration-dependent in both channels. As expected, FAD was detected only in the red channel with a lifetime of approximately 2 ns (Fig. 2b), in agreement with the previously reported values^30^. Signal originating from keratin in solution was present at all excitation wavelengths with an average lifetime of 1.5 ns, as reported previously^21^. Similar fluorescence lifetime values were acquired from structural keratin in a hair sample (Supplementary material, Fig. S3), implying that fluorescence lifetime of keratin is consistent for different isoforms, as well as for the structural and unfolded protein. The emission from NADH and FAD when present in a mixture can be separated, as the short lifetime component of 0.5 ns corresponding to NADH dominates in the blue channel (Fig. 2a), and the longer 2 ns lifetime from FAD is detected in the red channel (Fig. 2b). Interestingly, the combined keratin and NADH solution shows an excitation-wavelength dependent shift which differs between the channels at 780 nm, with a shorter lifetime component of 0.5 ns at 725–750 nm for both channels, increasing at 780 nm to 0.7 ns in the blue channel (445/60 nm) and to 1.5 ns in the red channel (580/150 nm), concluding at 1.5 ns at 800–810 nm in both spectral channels (Fig. 2a,b). This shift in the fluorescence lifetime implies that the contribution of NADH and keratin to the amplitude weighted average lifetime fluctuates depending on the excitation region, and on the spectral channel. This shift will be of importance when interpreting the MPM-FLIM of live keratinocytes.

While this study discusses the MPM-FLIM interpretation in the context of metabolic molecules and keratin, it is important to note that other proteins will exhibit 2PE intrinsic fluorescence, mostly due to the presence of aromatic amino acids—phenylalanine, tyrosine and tryptophan^13^. The emission spectra and fluorescence lifetime of tryptophan in proteins are dependent on the polarity and local environment^13^. This suggests that proteins may exhibit different spectral and lifetime characteristics based on not only their primary structure but also on their immediate environment. Thus, the autofluorescence observed when using MPM-FLIM will be a consequence not only of metabolic molecules but also the complex array of proteins present. Although keratin as an abundant component of keratinocytes is central to this study, to illustrate that proteins generally will contribute to fluorescence, we assessed the spectral characteristics of bovine serum albumin. This confirms that other proteins can be excited using two-photon excitation, and will exhibit both a spectral emission, as well as a fluorescence lifetime profile (τ_BSA_ ≈ 2 ns) (BSA, Fig. S4).

The average fluorescence lifetime values collected on our experimental system from the fluorophores in solution and hair, are presented in Table 2. Taken together, these results indicate that the differentiation between NADH, FAD, and keratin is possible through the investigation of fluorescence lifetime distribution histograms (Figs. S1–S3) and average fluorescence lifetimes (Fig. 2) in the chosen excitation wavelength range and with the corresponding spectral filters (blue 445/60 nm, red 580/150 nm). Although the investigation of one- and two-component solutions provides relatively transparent results, recognition, and identification of those fluorophores in a complex cellular environment will be more challenging.

**Table 2:** Fluorescence lifetime data from the measurements done on the fluorophores in solution, and hair.

	NADH 150 µM	NADH 5000 µM	FAD 100 µM	NADH 150 µM/FAD 100 µM	Keratin 180 µM	Hair (structural keratin)	NADH 150 µM/keratin 180 µM
725 nm	0.5 ns^a,b^	0.5 ns^a,b^	2.0–2.5 ns^b^	0.5 ns^a^ 2.0–2.5 ns^b^	1.5 ns^a,b^	1.5 ns^a,b^	0.5 ns^a,b^
750 nm	0.5 ns^a,b^	0.5 ns^a,b^	2.0–2.5 ns^b^	0.5 ns^a^ 2.0–2.5 ns^b^	1.5 ns^a,b^	1.5 ns^a,b^	0.5 ns^a,b^
780 nm	0.5 ns^a,b^	0.5 ns^a,b^	2.0–2.5 ns^b^	0.5 ns^a^ 2.0–2.5 ns^b^	1.5 ns^a,b^	1.5 ns^a,b^	0.7 ns^a^ 1.5 ns^b^
800 nm	–	0.5 ns^a,b^	2.0–2.5 ns^b^	0.5 ns^a^ 2.0–2.5 ns^b^	1.5 ns^a,b^	1.5 ns^a,b^	1.5 ns^a,b^
810 nm	–	0.5 ns^a,b^	2.0–2.5 ns^b^	0.5 ns^a^ 2.0–2.5 ns^b^	1.5 ns^a,b^	1.5 ns^a,b^	1.5 ns^a,b^

^a^445/60 nm channel.

^b^580/150 nm channel.

### Keratinocytes and fibroblasts exhibit the same spectral shift

**Figure 3:**
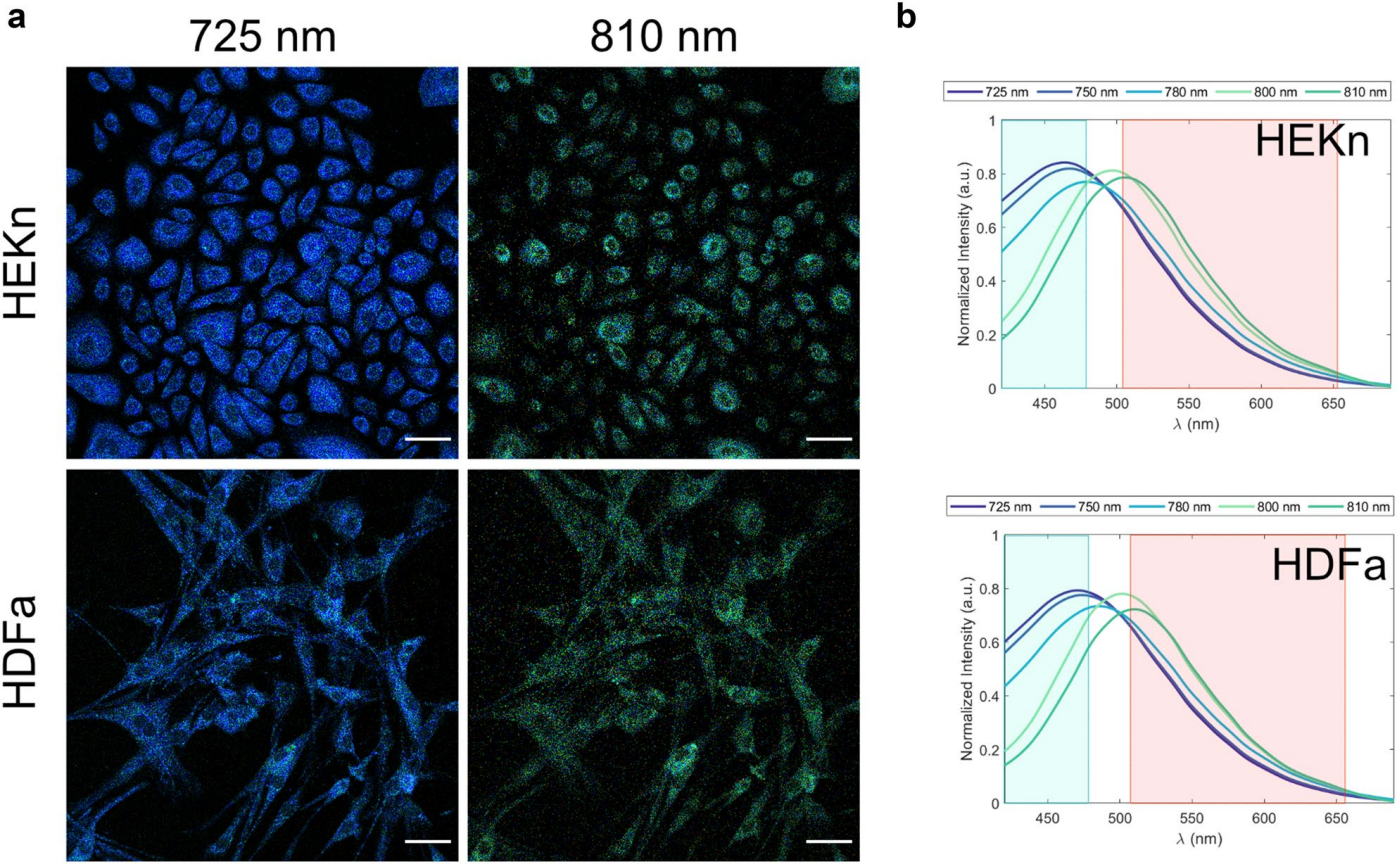
Human keratinocytes and human fibroblasts exhibit the same excitation-dependent spectral shift in the emission spectrum. (**a**) Multiphoton spectral imaging of human keratinocytes (HEKn) and human fibroblasts (HDFa) collected with a spectral detector in the emission range of 416–683 nm at the excitation wavelength of 725 nm and 810 nm. Scale bar: 50 µm. The brightness in the images has been adjusted for clarity. (**b**) Emission spectra extracted from the images at the excitation wavelength: 725, 750, 780, 800, and 810 nm for keratinocytes and fibroblasts.

Spectral lambda images and spectral emission data were acquired from human keratinocytes (HEKn) and human fibroblasts (HDFa) using MPM with a spectral detector. Lambda images from all excitation wavelengths together with the emission spectra are presented in the Supplementary material (Figs. S5 and S6). The characteristic epithelial morphology for keratinocytes (Fig. 3a), and the elongated spindle morphology for fibroblasts (Fig. 3b) are visible, suggesting a proper adhesion and cell migration for both cell types. The spectral data in both cell types displays a shift in the emission from blue to green with the increase of the excitation wavelength from 725 to 810 nm, seen as well as a change in the lambda coded color-scale from dark blue to green. The alteration in emission suggests that NADH (blue region) dominates at shorter excitation wavelengths, while the contribution from FAD is expected to intensify as the excitation wavelength is increased, in agreement with the spectral measurements (Figs. 1 and 2 and Table 2) and literature data (Table 1). Even though the intrinsic fluorophore composition is expected to differ between the cell types, the acquired spectral data did not show any significant differences between keratinocytes and fibroblasts. Instead, the emission spectra were similar in the chosen range of excitation wavelength indicating complex spectral crosstalk, possibly arising from several different intrinsic fluorophores, which are likely to contribute to the emission.

### FLIM analysis reveals differences in lifetime distribution between keratinocytes and fibroblasts

**Figure 4:**
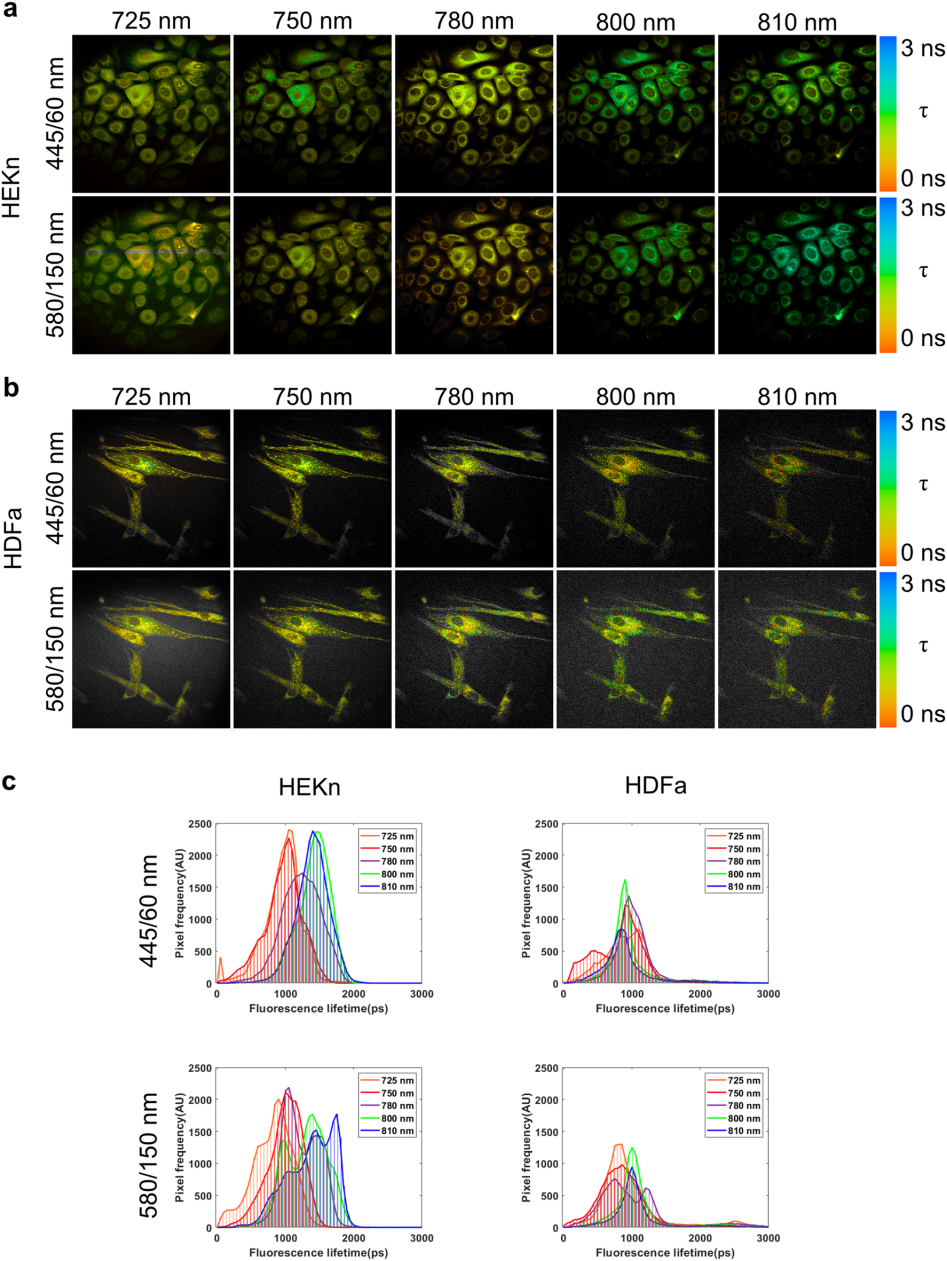
MPM-FLIM analysis reveals a longer fluorescence lifetime component of 1.5 ns present in human keratinocytes, but not in human fibroblasts. MPM-FLIM images of (**a**) human keratinocytes and (**b**) human fibroblasts collected in the blue (445/60 nm) and red (580/150 nm) channel, (**c**) and the corresponding lifetime distribution histograms recorded in the excitation range of 725 to 810 nm. Field of view: ~ 350 × 350 µm^2^. False-color scale fluorescence lifetime data, 256-time channels, ranging from 0 to 3 ns. Brightness and contrast have been adjusted for clarity. The lifetime distribution histograms represent the average lifetime distribution acquired from the whole image frame from three biological replicates.

MPM-FLIM images and corresponding fluorescence lifetime distribution histograms were acquired from human keratinocytes (HEKn) and compared with human fibroblasts (HDFa) in the blue (445/60 nm) and the red (580/150 nm) channels (Fig. 4). An excitation-dependent shift in the amplitude weighted average lifetime distribution was observed in keratinocytes in both channels, from approximately 1 ns to 1.5 ns (Fig. 4a,c) with the increased excitation wavelength. In contrast, the overall fluorescence lifetime values for the fibroblasts were shorter, in the range 0.5 ns to 1 ns, and did not exhibit the same clear excitation dependence (Fig. 4b,c). The observed shift in keratinocytes resembles the shift observed for NADH and keratin mixture (Fig. 2, Fig. S2), leading us to speculate that the high abundance of keratin, seen in keratinocytes^37^, may influence the MPM-FLIM reading in this cell type, and should therefore be further scrutinized. A longer fluorescence lifetime component (≥ 1 ns) has been previously reported as originating from enzymatically bound NADH^35,38–40^. However, the appearance of the lifetime component at 1.5 ns, prominent in keratinocytes but not in fibroblasts, which according to the previous studies^39,40^ would be interpreted as enzymatically bound NADH, suggests that the detected lifetime originates from another intrinsic fluorophore, present only in keratinocytes. Even though the cell types may differ metabolically, according to our measurements on NADH and keratin in solution (Fig. 2, Fig. S2), keratin is the main contributor to the detected average fluorescence lifetime in the excitation range of 780 to 810 nm, therefore the effect of cell metabolism in this excitation wavelength window would be minimal. Instead, the metabolic differences would be observed in the excitation wavelength range of 725 to 750 nm.

MPM-FLIM images acquired in both channels (blue 445/60 nm and red 580/150 nm), together with fluorescence lifetime distribution histograms in the range of 0 to 5 ns and more detailed fluorescence lifetime distribution, are presented in the Supplementary material (Figs. S7–S12).

### Keratin depletion leads to significant FLIM changes

Keratin has been reported as an important skin fluorophore^20,21,24^, and comprises 17–27% of total cellular proteins in a basal keratinocyte^37^. Even though the data on spectral keratin characterization are rather sparse, and the excitation process is not well understood, keratin may influence MPM-FLIM imaging. Therefore, to investigate the effect of keratin on the MPM-FLIM readout, we have further examined the observed cellular autofluorescence and performed siRNA experiments to reduce the keratin content in keratinocytes.

**Figure 5:**
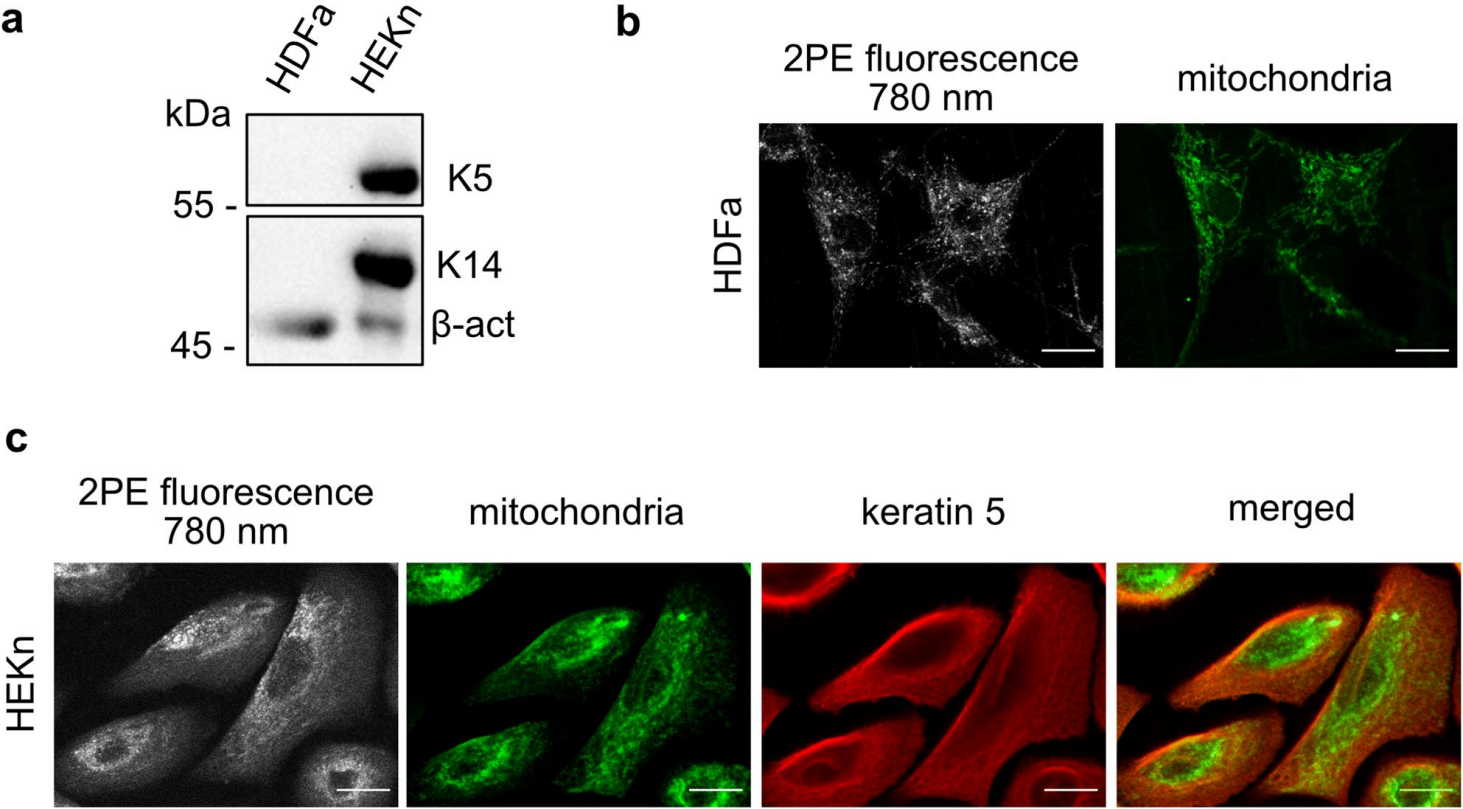
The overlap of keratin intermediate filaments with the mitochondria in keratinocytes makes it challenging to separate the 2PE mitochondrial and keratin signal. Intrinsic cellular fluorescence of keratinocytes and fibroblasts investigated with respect to keratin levels, and immunostaining. (**a**) Levels of keratin 5 and keratin 14 in keratinocytes and fibroblasts analyzed by Western blotting. The membrane was cut in half prior to the antibody probing. The membrane containing proteins larger in size than 55 kDa was probed with an anti-cytokeratin 5 antibody, followed by an incubation with a secondary antibody. The membrane containing proteins smaller in size than 55 kDa was probed simultaneously with anti-cytokeratin 14 and anti-β-actin antibody, followed by simultaneous incubation with the secondary antibodies. The membranes were imaged with the same exposure time (2 min). Protein loadings were standardized for total protein content based on Coomassie blue stained gels and densitometric analysis. An antibody to β actin was used on the same membrane to demonstrate even loading. (**b**) Intensity MPM image of fibroblasts excited at 780 nm collected with GaAsP detector on an LSM 710 NLO microscope. Cells were fixed and stained for mitochondria with an antibody to ATP5A after the label-free MPM imaging. Stained fibroblasts were then imaged on the same system using a confocal module and an Ar-laser at 488 nm. (**c**) Intensity MPM image of keratinocytes excited at 780 nm collected with GaAsP detector on an LSM 710 NLO microscope, which were fixed and stained for keratin 5 with an antibody, and mitochondria with an ATP5A antibody, after the label-free MPM imaging. Stained keratinocytes were then imaged on the same system using a confocal module and an Ar-laser 488 nm (mitochondria) and Laser diode 561 nm (keratin 5). Images were merged to visualize mitochondrial and keratin co-localization. Scale bar: 10 µm. Brightness and contrast have been adjusted for clarity.

Western blotting was performed to confirm the presence of keratin isoforms, and immunostaining for mitochondria and keratin 5 was done in combination with MPM imaging to facilitate a better understanding of the intracellular localization of the anticipated intrinsic fluorophores (Fig. 5). As expected, the Western blot analysis confirmed the presence of both keratin 5 and keratin 14 in keratinocytes, which were not present in fibroblasts (Fig. 5a, Fig. S13). The MPM images collected at 780 nm excitation reveal that both cell types, i.e., HDFa (Fig. 5b) and HEKn (Fig. 5c), show dense fluorescent spindle-shaped structures spanning the cell body, co-localizing with the mitochondrial staining, suggesting that the intrinsic fluorescence of this structure originates from the mitochondrial components. In addition, a more diffuse fluorescence originating from the cytoplasmic area was observed, overlapping with the spindle-shaped structures in the keratinocytes. This cytoplasmic signal was dependent on the excitation wavelength in fibroblasts and diminished at 780 nm (Supplementary material, Fig. S14); however, the cytoplasmic signal in keratinocytes was still present at 780 nm, suggesting the presence of an additional fluorophore, not present in fibroblasts. This structure was found to correlate with the keratin 5 staining, suggesting that keratin filaments may contribute to the intrinsic cellular fluorescence when performing MPM in keratinocytes (Fig. 5c). Furthermore, the mitochondrial and keratin staining shows a significant overlap suggesting that, if keratin contributes to the FLIM imaging, the separation of the mitochondrial and keratin signals will be challenging.

**Figure 6:**
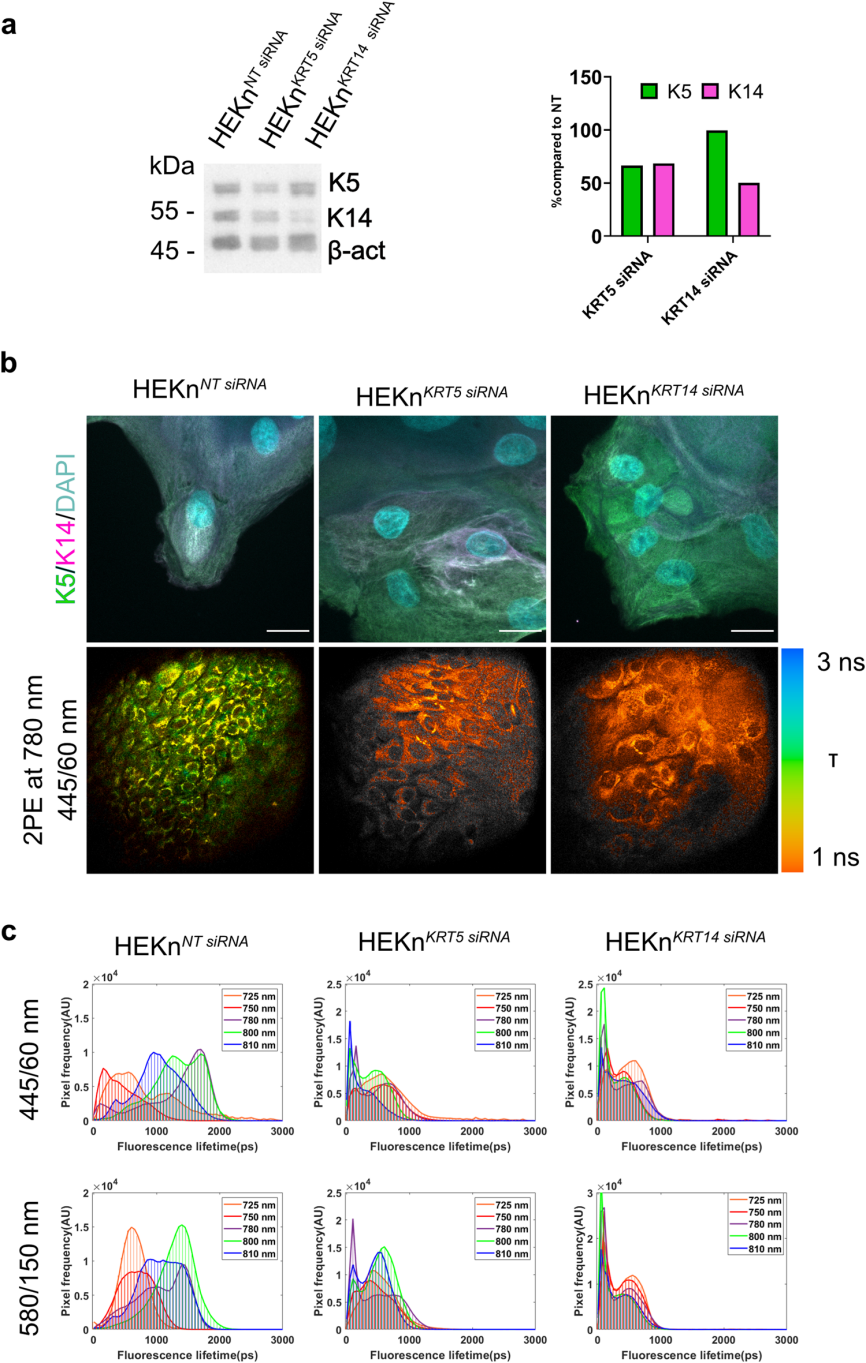
Longer fluorescence lifetime component of 1.5 ns is not detected in keratin-depleted keratinocytes. (**a**) Depletion of keratin 5 and keratin 14 analyzed by Western blotting where non-targeting siRNA samples are used as a control. The primary antibodies were incubated simultaneously, as were the secondary antibodies. Protein loadings were standardized for total protein content based on Coomassie blue stained gels and densitometric analysis. An antibody to β actin was used on the same membrane to demonstrate even loading. (**b**) Upper panel: confocal images of HEKn^NT siRNA^, HEKn^KRT5 siRNA^ and HEKn^KRT14 siRNA^ keratinocytes stained for keratin 5, keratin 14 and DAPI. Images were merged to visualize keratin 5 and keratin 14 co-localization. Scale bar: 10 µm. Lower panel: MPM-FLIM color-coded images of HEKn^NT siRNA^, HEKn^KRT5 siRNA^ and HEKn^KRT14 siRNA^ in the range of 1 to 3 ns recorded in blue channel (445/60 nm). Field of view: ~ 350 × 350 µm^2^. False-color scale fluorescence lifetime data, 256-time channels, ranging from 1 to 3 ns. Brightness and contrast have been adjusted for clarity. (**c**) Fluorescence lifetime distribution histograms recorded for HEKn^*NT siRNA*^, HEKn^*KRT5 siRNA*^ and HEKn^*KRT1 siRNA-*^ keratinocytes in the range of 0 to 3 ns in the blue (445/60 nm) and red (580/150 nm) channel in the excitation range of 725 to 810 nm. False-color scale fluorescence lifetime data, 256-time channels, ranging from 0 to 3 ns.

To further challenge the possible contribution of keratin to the fluorescence signal in MPM-FLIM imaging of keratinocytes, an siRNA silencing experiment was performed to deplete keratin 5 and keratin 14 in keratinocytes. As discussed previously, the amplitude weighted fluorescence lifetime will depend on the local concentration of the fluorophores contributing to the fluorescence signal. Thereby, if keratin contributes to the fluorescence lifetime data, a perturbation of the cellular concentration of keratin is expected to lead to changes in the fluorescence lifetime distribution. SiRNA silencing (96 h) of KRT5 led to approximately 40% depletion of keratin 5 and a simultaneous 40% depletion of keratin 14, whilst siRNA silencing of KRT14 led to approximately 50% depletion of keratin 14, as compared to the non-targeting (NT) siRNA sample (Fig. 6a, Fig. S15). Fluorescence images of cells following depletion of keratin, stained for keratin 5 and keratin 14, did not show any significant disruption of the keratin filament network (Fig. 6b, Fig. S16–S17). However, the MPM-FLIM data revealed a distinct shift of the amplitude weighted fluorescence lifetime towards shorter lifetimes in the KRT5 and KRT14 silenced keratinocytes compared to NT siRNA, seen as a change from green to orange in the color coded MPM-FLIM images (here at 780 nm excitation). This change in fluorescence lifetime distribution was confirmed for the whole excitation range in both spectral channels (Fig. 6c) and supports the hypothesis that the 1.5 ns fluorescence lifetime observed in the fluorescence lifetime distribution histograms of keratinocytes most likely relates to keratin (see additionally Figs. S18–S26 for detailed MPM-FLIM analysis).

The observed shift in fluorescence lifetime in keratin-depleted keratinocytes may be a result of the decrease in the local keratin concentration, which would influence the FLIM signal detection. However, changes in fluorescence lifetime may occur due to local microenvironment changes, such as pH^34^ or viscosity^41^. Therefore, the observed fluorescence lifetime changes may be an effect of the protein depletion on the local environment itself. Certainly, the siRNA silencing may lead to the changes in the metabolism, which would have affected the fluorescence lifetime distribution in the excitation wavelength range of 725 to 750 nm; however, as seen previously (Fig. 2, Fig. S2), keratin is the main contributor to the signal in the range of 780 to 810 nm. Therefore, the effect of change in the cell metabolism in this wavelength window would be minimal, especially in the red channel (580/150 nm). While we cannot confirm that keratin is the main source of fluorescence in keratinocytes in the range 780 to 810 nm, our data support that keratin has an effect on the MPM-FLIM readout and could therefore be incorrectly assigned as NADH in the studies of cellular redox ratio.

## Conclusions

The possibility to visualize tissue morphology based on the intrinsic cellular fluorescence is beneficial when targeting the development of non-invasive and label-free imaging technologies. The intrinsic fluorescence in the context of MPM has been seen as valuable, as it enables high resolution, non-invasive imaging of live cells and tissues. In combination with fluorescence lifetime imaging, MPM-FLIM has been proposed to provide data revealing information about the metabolic state of the tissue; however, intrinsic fluorescence is complex. In this study, we scrutinize the spectral properties of keratinocytes and interpret the findings in the context of cellular localization and composition. We show that the proteins can be excited with 2PE in the range of 725 to 810 nm and may contribute to the MPM-FLIM signal. We identify keratin as a potential contributor to the observed fluorescence lifetime of 1.5 ns in keratinocytes in the excitation wavelength range of 780 to 810 nm, as the depletion of keratin 5 and keratin 14 affects the MPM-FLIM readout. This finding is of high importance, particularly when combining epidermal tissue cultures and keratin-rich specimens with bioimaging. Whilst the fluorescence of other endogenous fluorophores in the tissue is often neglected, keratin together with other proteins exhibiting intrinsic fluorescence may contribute to the fluorescence lifetime signal in MPM-FLIM, and could therefore be mistaken for NADH or FAD due to the spectral properties, leading to the misinterpretation of cellular redox ratio^16^ in the MPM-FLIM studies of, e.g., skin, if the incorrect excitation wavelength is chosen. We propose that for the metabolic imaging of skin and keratin-containing samples, NADH should be excited in the range of 720 to 750 nm (emission collected at ≈ 470 nm), and FAD at 900 nm (emission collected at > 510 nm), to avoid keratin crosstalk. The excitation wavelength range of 780 to 810 nm should be used with caution, unless the imaging of keratin is of interest. Taken together, our results suggest that the interpretation of MPM-FLIM data originating from cellular autofluorescence is far more complex than previously suggested and that other tissue constituents should not be neglected—changing the paradigm for data interpretation in this context.

## Materials and methods

### Fluorophores in solution

NADH (10107735001, Roche) was reconstituted to give a stock solution of 5 mM in 0.01 N NaOH, and FAD (F6625, Sigma) stock solution was prepared at the concentration of 5 mM in MilliQ water. The NADH and FAD working solutions were then prepared in PBS, pH 7.4, at concentrations 150 µM and 100 µM, respectively, or as a mixture of both.

Human keratin solution (K0253, Sigma) at a concentration of 11 mg protein/ml was used directly for imaging. If one assumes the keratin mass to be 62 kDa, this corresponds to a concentration of approximately 180 µM. NADH and keratin mixture was prepared by mixing keratin solution (180 µM) with NADH solution (750 µM) in ratio 4:1, giving a final concentration of keratin 140 µM and 150 µM for NADH.

Bovine serum albumin (A7030, Sigma) was dissolved in 8 M urea, 50 mM Tris, 0.1 M β-mercaptoethanol, pH 8.4 at a concentration 180 µM.

### Hair

Light blonde hair was obtained from a female volunteer and mounted on a microscope slide in PBS, pH 7.4, with a coverslip (#1.5).

### Cell culture

Neonatal human epidermal keratinocytes (HEKn) and adult human dermal fibroblasts (HDFa) were from Thermo Fisher Scientific.

HEKn cells were cultured in Epilife^®^ (Gibco™) growth medium supplemented with 60 µM of Ca^2+^, Human Keratinocyte Growth Supplement (HKGS, Gibco™), and Gentamicin/Amphotericin (G/A, Gibco™). The addition of HKGS to the growth medium provides 0.2% bovine pituitary extract, 1 μg/mL recombinant human insulin-like growth factor-I, 0.18 μg/mL hydrocortisone, 5 μg/mL bovine transferrin, and 0.2 ng/mL human epidermal growth factor. The cells were seeded at a density of 2.5 × 10^3^ cells/cm^2^ in T-25 flask in 5 ml of supplemented growth medium and incubated in a humified cell culture incubator at 37 °C in 5% CO_2_ and 95% air. The growth medium was changed every other day until 50% confluence and every day thereafter. The cells were subcultured at 80% confluence.

HDFa cells were cultured in Human Fibroblast Expansion Basal Medium (HFEBM, Gibco™) supplemented with Low Serum Growth Supplement (LSGS, Gibco™), and Gentamicin/Amphotericin (G/A, Gibco™). The addition of LSGS to the growth medium provides 2% fetal bovine serum, 1 μg/mL hydrocortisone, 10 ng/mL human epidermal growth factor, 3 ng/mL basic fibroblast growth factor, and 10 µg/mL heparin. The cells were seeded at the density of 5 × 10^3^ cells/cm^2^ in T-25 flask in 5 ml of supplemented growth medium and incubated in a humified cell culture incubator at 37 °C in 5% CO_2_ and 95% air. The growth medium was changed every other day until 50% confluence and every day thereafter. The cells were subcultured at 80% confluence.

For spectral and MPM-FLIM imaging, the cells were seeded on a glass bottom dish (ibidi, #1.5 mm, Ø = 35 mm) at a seeding concentration of 1 × 10^4^ cells/cm^2^ and grown for 48 h. On the day of the imaging, the cells were washed twice in warm PBS complete (PBS, 1 mM CaCl_2,_ 0.5 mM MgCl_2_) and imaged in fresh PBS complete solution. Imaging was performed at 37 °C in a humidity chamber.

### Protein extraction and western blotting

Cells were grown until 80% confluence and detached with 0.05% Trypsin/EDTA solution (Gibco™) at RT. Trypsin was then neutralized by the addition of Trypsin Neutralizer (Gibco™) and centrifuged at 150*g* for 7 min at 17 ºC. Cells were resuspended in ice-cold PBS and centrifuged at 150*g* for 7 min. at 4 ºC. Cells were then lysed in ice-cold lysis buffer (50 mM HEPES at pH 7.2, 90 mM KCl, 0.5% Igepal) containing Protease Inhibitor Cocktail (P8340, Sigma) and centrifuged at 7000 rpm at 4 ºC for 5 min in a benchtop centrifuge. The post nuclear supernatant was stored at −20 ºC until analysis. Samples were diluted in SDS sample buffer, heated for 2 min. at 96 ºC and resolved on a 10% acrylamide gel. For standardization of protein levels, gels were stained with Coomassie blue, and lane intensities quantified using ImageJ^42^. Following loading adjustments to equalize protein levels, resolved proteins were transferred to nitrocellulose membranes overnight using a wet transfer system.

Nitrocellulose membranes were briefly stained with Ponceau stain, washed in 0.1% PBS-Tween, and blocked in 5% milk in PBS for 30 min. Membranes were then stained for 1 h with primary antibodies diluted in blocking milk, washed three times in 0.1% PBS-Tween for 5 min., followed by a 1 h incubation with secondary antibodies diluted in blocking milk. The protein bands were quantified with ImageJ^42^. Primary antibodies were Anti-Cytokeratin 5 (ab52635, Abcam) and Anti-Cytokeratin 14 (ab7800, Abcam). Secondary antibodies were Anti-Mouse IgG (whole molecule, A4416, Sigma) and Anti-Rabbit IgG (whole molecule, A6154, Sigma).

### siRNA silencing

HEKn cells were seeded at a density of 2.5 × 10^3^ cells/cm^2^ in six-well plates with a glass bottom (#1.5, Cellvis) and grown for 48 h. One coverslip (10 mm, #1.5) was placed on the bottom of each well for immunofluorescence. siRNA silencing of KRT5 and KRT14 was carried out using Dharmacon™ Accell™ siRNA reagent with a SMARTPool format (Horizon Discovery), which contains a mixture of four siRNAs:

KRT5-1: GCUCCAGCGUCAAAUUUGU

KRT5-2: GUAGUGGAUUUGGUUUCGG

KRT5-3: CCACAUUCUUUGGUUCCCA

KRT5-4: GGUUGAUGCACUGAUGGAU

KRT14-1: CUGCUGAGAUCAAAGACUA

KRT14-2: GUAUGAGACAGAGUUGAAC

KRT14-3: UGCAGAACCUGGAGAUUGA

KRT14-4: UGGAUGUGCACGAUGGCAA

For one culture well (9.6 cm^2^), Accell™ siRNA was added at a final concentration of 1 µM, and the cells were incubated for 96 h. Two controls were used, HEKn cells grown in Accell Delivery Growth Medium supplemented with HKGS, and HEKn cells incubated with non-targeting Accell™ siRNA reagent, which contained a mixture of 4 siRNAs:

NT-1: UGGUUUACAUGUCGACUAA

NT-2: UGGUUUACAUGUUUUCUGA

NT-3: UGGUUUACAUGUUUUCCUA

NT-4: UGGUUUACAUGUUGUGUGA

After 96 h, the cells were imaged with MPM-FLIM (“Multiphoton laser scanning microscopy with fluorescence lifetime modality”), the coverslips were collected for immunofluorescence (“Immunofluorescence”), and the cell lysate was collected for protein extraction (“Protein extraction and western blotting”).

### Immunofluorescence

The procedure was carried out at room temperature. Cells grown in a glass bottom dish with a grid (ibidi, #1.5 mm, Ø = 35 mm) were washed three times in warm PBS complete, fixed in 4% formaldehyde in PBS for 10 min and then permeabilized in 0.2% Triton X100 in PBS for 1 h. The cells were then blocked in 3% bovine serum albumin (BSA) diluted in PBS for 30 min. and incubated for 1 h in a primary antibody diluted in 3% BSA, followed by three washes in PBS, and 1 h incubation with a corresponding secondary antibody diluted in 3% BSA. The cells were then washed three times in PBS, once in MilliQ water, and mounted with 10 µl of Prolong Gold and a coverslip (#1.5). The samples were allowed to set overnight at room temperature and stored at 4ºC in the dark thereafter. Primary antibodies were Anti-Cytokeratin 5 (ab52635, Abcam), Anti-ATP5A (Mitochondrial Marker, ab14748, Abcam) and Anti-Cytokeratin 14 (ab7800, Abcam). Secondary antibodies were Goat Anti-Rabbit IgG H&L Alexa Fluor^®^ 488 (ab150077, Abcam) and Goat Anti-Mouse IgG H&L AlexaFluor^®^ 597 (ab150116, Abcam).

### Microscopy

#### Confocal and spectral laser scanning microscopy

A commercial LSM 710 NLO (Carl Zeiss, Jena, Germany) was used for confocal imaging and spectral MPM. A water-dipping objective lens, Water Plan-Apochromat 20× (NA 1.0) was utilized for live cell imaging. The objective was dipped directly in the dish with the cells grown on the tissue culture-treated plastic bottom. For the spectral MPM-imaging, a fs-pulsed NIR laser (InSight Deepsee, Newport Spectra-Physics) was used, where power was kept at approximately 6 mW, as measured at the sample. For MPM imaging, a GaAsP detector with no spectral selection was used (apart from laser cut-off). For spectral imaging, the QUASAR spectral detector was used (421–693 nm, 34 channels, 9 nm resolution). Excitation spectra were acquired automatically tuning the InSight lasers in the range 680–810 nm.

For immunofluorescence imaging, the same microscope was used in the confocal mode. An Ar-laser (488 nm) was utilized for Alexa 488 conjugated secondary antibodies (493–630 nm emission), and a laser diode (561 nm) for Alexa 594 nm (585–733 nm emission). For imaging of the immunostained samples, an oil-immersion objective Plan-Apochromat 40× (NA 1.4) was used.

#### Multiphoton laser scanning microscopy with fluorescence lifetime modality

MPM-FLIM was performed using an experimental MPM inverted system, as described in the previous work^43^. Cells were imaged using glass bottom dishes (ibidi, #1.5, Ø = 35 mm). A fs-pulsed NIR laser (Tsunami laser, Newport Spectra-Physics) was set to operate at five excitation wavelengths: 725 nm, 750 nm, 780 nm, 800 nm, and 810 nm. The selection of excitation wavelength was limited to 810 nm due to the performance issue of the laser. The pulse width dispersion at the sample plane was controlled to be ~ 100 fs for all wavelengths, using an autocorrelator (CARPE, APE) and pulse compressor (Femto control kit, APE). The laser power was kept at approximately 20 mW, as measured at the sample, controlled by a Pockels cell (350-80LA, ConOptics). A water immersion objective (40× , 0.8 C "Achroplan" NIR, Carl Zeiss) was used. The detection was performed by two separate GaAsP detectors (H7422P-40 MOD, Hamamatsu), interfaced to two time-correlated single-photon counting modules (SPC 150, Becker & Hickl), enabling FLIM acquisition. Two spectral combinations were enabled by utilizing the dichroic mirror (509 nm cut off, Semrock Inc) combined with filter combinations in red 580/150 nm and blue 445/60 nm (Semrock, Brightline). The data acquisition time for FLIM is approximately 60 s for each image plane.

### Image processing and data analysis

Image processing and spectral analysis were done using ImageJ^42,44^ (U.S. National Institutes of Health, Bethesda, Maryland) and MatLab (vR2020b, MathWorks Inc). MPM intensity images data were created by SPCM64 (Becker & Hickl) software and MPM-FLIM images were analyzed by SPCImage software (v 9.82, Becker & Hickl). The instrument response function (IRF) was approximately 200 ps using the automated IRF setting in the software. A multiexponential decay fitting model was used for FLIM data analysis, having a time window of 12.5 ns corresponding to the repetition rate of the laser. The system was calibrated by measuring the fluorescence lifetime of Rhodamine B solution, detected at 1.3 ns, in good agreement with the literature^45^.

A single exponential decay model was used for modeling fluorescence decay obtained from intrinsic fluorophores in solutions,

1 $$f\left(t\right)={e}^{-\mathrm{t}/\tau }$$

where $f\left(t\right)$ is fluorescence decay and $\tau$ is fluorescence lifetime. A biexponential decay model was chosen for all the data obtained from the cellular samples and two-component solutions,

2 $$f\left(t\right)={{a}_{1} e}^{-\mathrm{t}/{\tau }_{1}}+{{a}_{2} e}^{-\mathrm{t}/{\tau }_{2}}$$

where ${\tau }_{1}$ corresponds to the fast and ${\tau }_{2}$ the slow lifetime component. The pre-exponential factors ${a}_{1}$ and ${a}_{2}$ describe the amplitude components of the fast and slow decay respectively. The model fitting was optimized with respect to keeping the reduced ${\chi }^{2}$ value as close to 1 as possible, indicating that the correspondence between observations and estimated model is in accordance with the error variance. The biexponential model was chosen over triexponential decay model because the reduced χ^2^ value did not improve significantly with the triexponential model (Supplementary Material, Fig. S27). Typically, χ^2^ values range between 0.95 and 1.50 was found to best fit the data. When fitting the data to an exponential decay, there will be a range of τ-values that potentially fit the data. Therefore, in order to make the assessment of FLIM data more robust, the distribution of the amplitude weighted average lifetime $({\tau }_{m})$ was primarily assessed, defined as

3 $${\tau }_{m}=\frac{{a}_{1}{\tau }_{1}+{a}_{2}{\tau }_{2}}{{a}_{1}+{a}_{2}}.$$

This parameter was found to generate more consistent data for repeated experiments, and the distribution of ${\tau }_{m}$ was plotted for different excitation and spectral wavelengths for the investigated cell samples. The range of ${\tau }_{m}$ between 0 and 5000 ps was assigned to false color scale (red to blue) to generate FLIM images and ${\tau }_{m}$ distributions were extracted. For a more in-depth analysis, the ${\tau }_{m}$ range was subdivided: 0–1000 ps, 1000–2000 ps, and 2000–5000 ps. Fluorescence lifetime data was extracted from the SPCImage software. The distribution graphs images were plotted in MatLab.

## Supplementary Information

Supplementary Figures.

## Data Availability

The datasets used and/or analyzed during the current study are available from the corresponding author on reasonable request.
