# Supplementary material for: Contribution of autofluorescence from intracellular proteins in multiphoton fluorescence lifetime imaging
Source: Sci Rep. 2022 Oct 5;12:16584. doi: 10.1038/s41598-022-20857-6 (PMC9534927; doi:10.1038/s41598-022-20857-6)
Supplement: Supplementary file 1 — Supplementary Figures. [file 41598_2022_20857_MOESM1_ESM.pdf]

Supplementary Material

Contribution of autofluorescence from intracellular proteins in multiphoton fluorescence lifetime imaging

Monika Malak,[a,*] Jeemol James,[a] Julie Grantham,[b] Marica B. Ericson[a,*]

[a] University of Gothenburg, Faculty of Science, Department of Chemistry and Molecular Biology, Biomedical photonics, Kemivägen 10, Gothenburg, Sweden, 412 96

[b] University of Gothenburg, Faculty of Science, Department of Chemistry and Molecular Biology, Medicinaregatan 9C, Gothenburg, Sweden, 413 90

Supplementary data

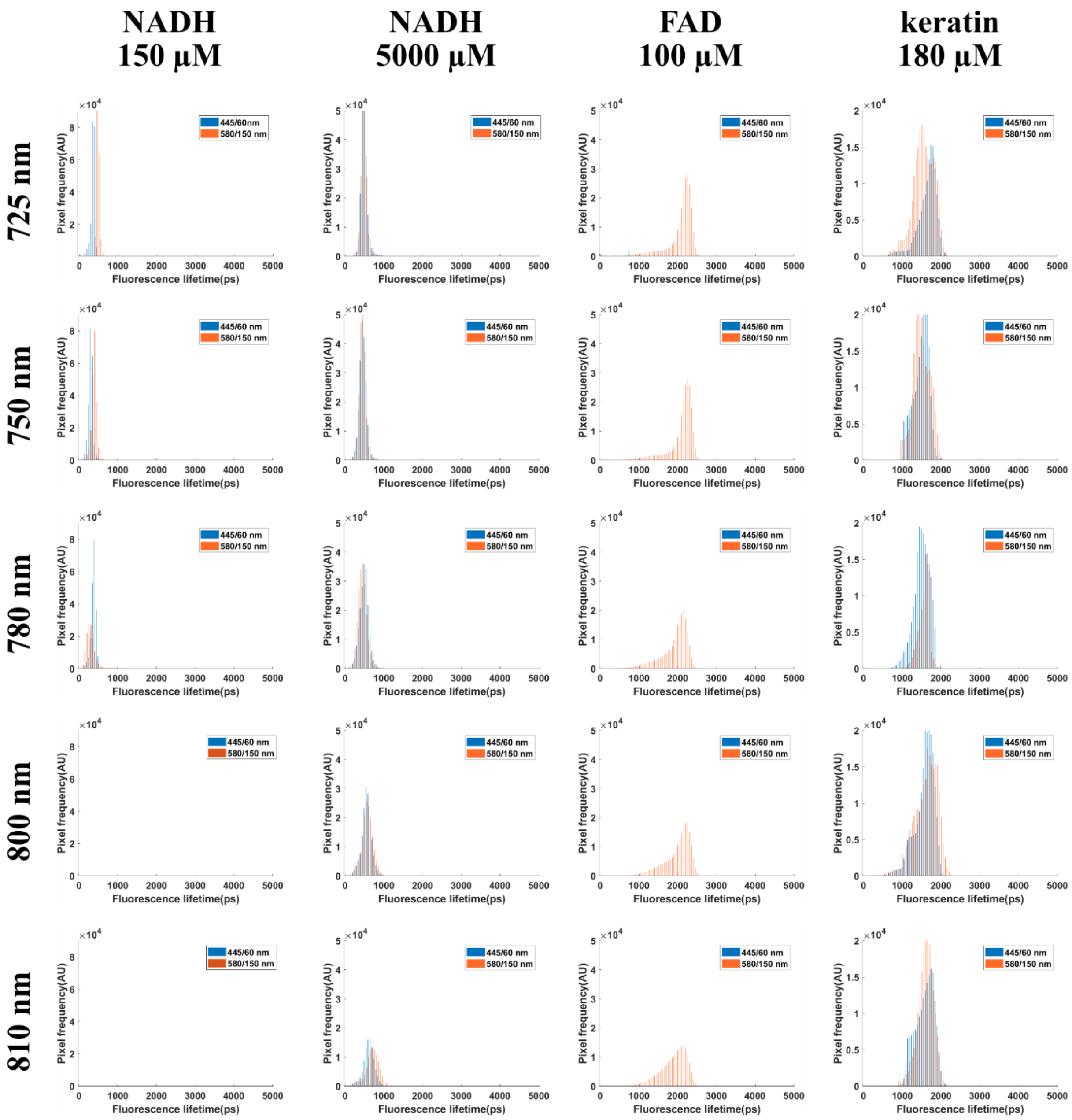

Fig. S1 Fluorescence lifetime detection of pure fluorophores in solution. Fluorescence lifetime distribution histograms of NADH (150 µM), NADH (5 mM), FAD (100 µM), and keratin (180 µM) in solution, collected in the blue (445/60 nm) and red (580/150 nm) channel in the excitation range of 725 – 810 nm. The fluorescence lifetime range has been adjusted to 0 to 5 ns.

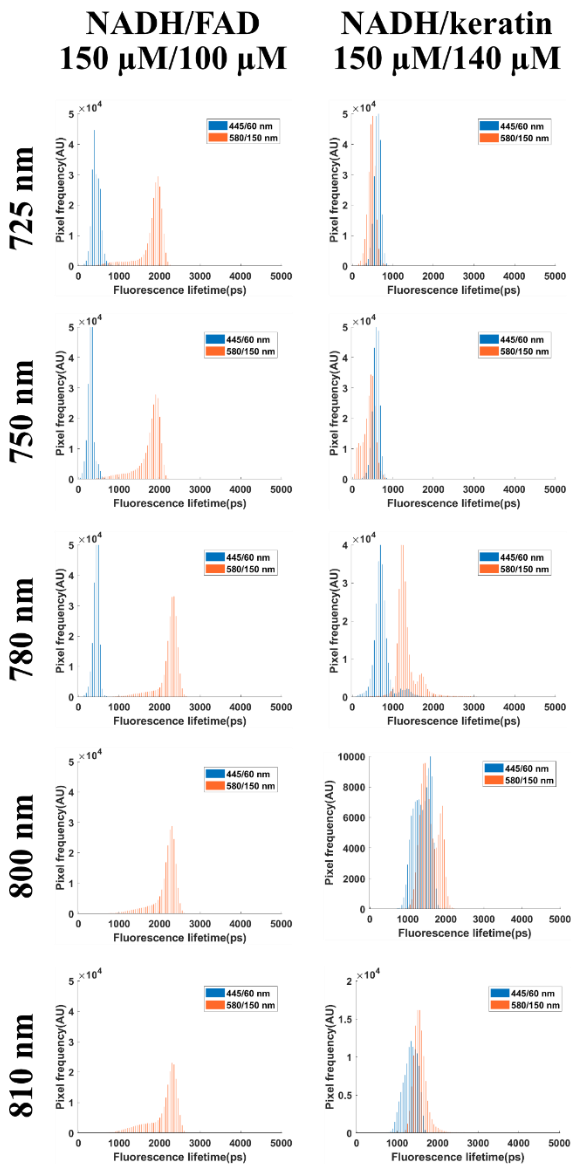

Fig. S2 Fluorescence lifetime detection of fluorophore mixtures in solution. Fluorescence lifetime distribution histograms of NADH/ FAD mixture (150/100 µM), and NADH/keratin mixture (150 µM/140 µM) in solution, collected in the blue (445/60 nm) and red (580/150 nm) channel in the excitation range of 725 – 810 nm. The fluorescence lifetime range has been adjusted to 0 to 5 ns.

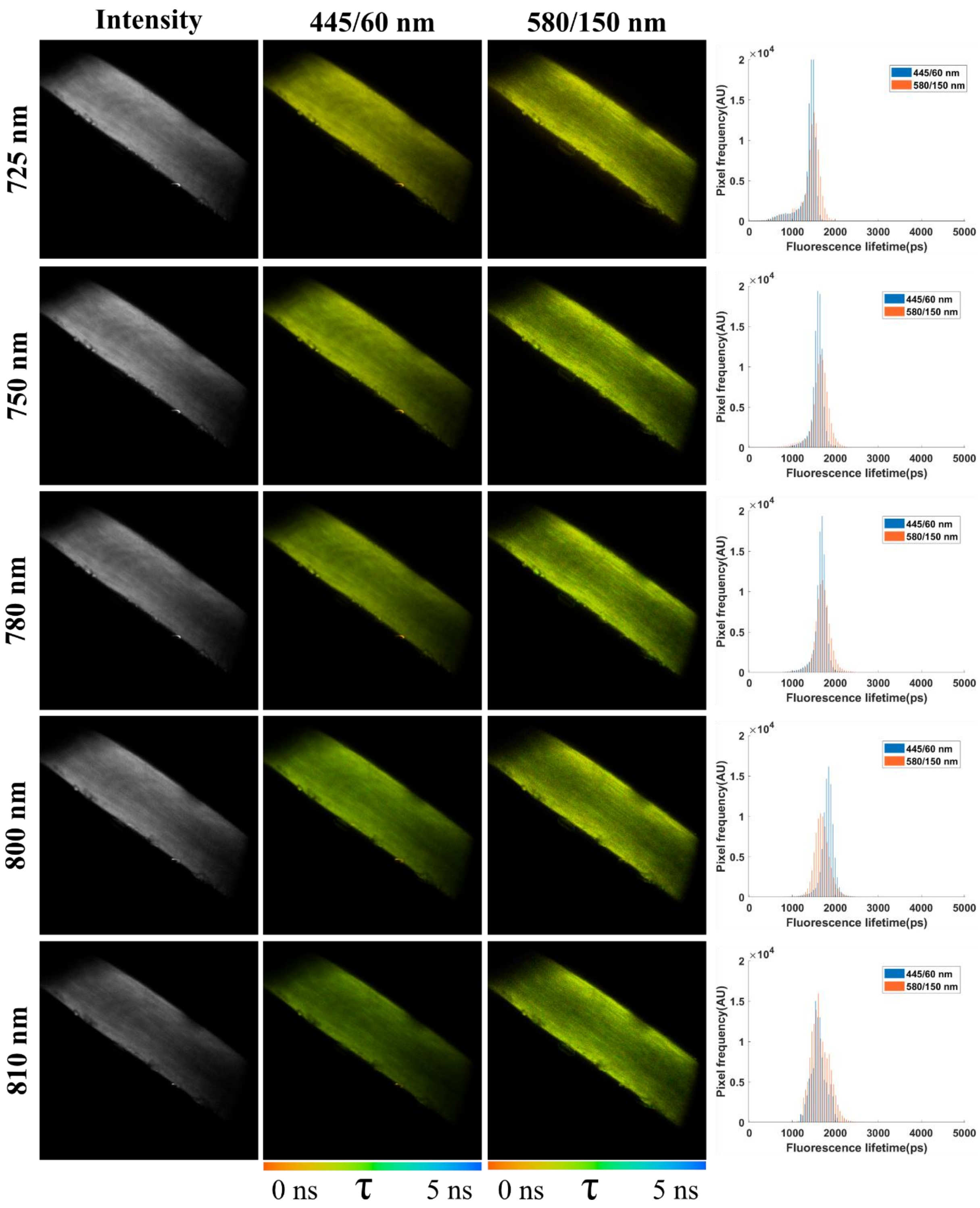

Fig. S3 MPM-FLIM imaging of hair – keratin-rich component. Intensity and MPM-FLIM images of human hair in the excitation range of 725 – 810 nm collected in the blue (445/60 nm) and red (580/150 nm) channel, and the corresponding lifetime histograms. The fluorescence lifetime range has been adjusted to 0 to 5 ns. Brightness and contrast in the images were adjusted for clarity. Field of view: ~ 350 x 350 μm^2. False-color scale fluorescence lifetime data, 256-time channels, ranging from 0 to 5 ns.

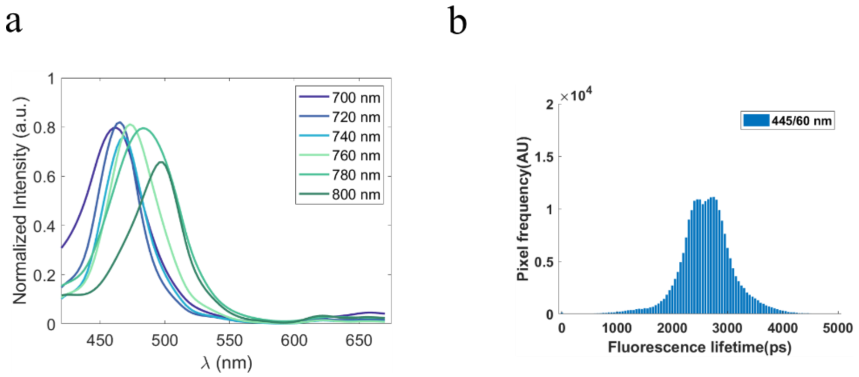

Fig. S4 Two-photon intrinsic fluorescence of bovine serum albumin in solution. Bovine serum albumin (BSA) was dissolved at the concentration of 11 mg/ml in a urea solvent (8M urea, 50 mM Tris, 0.1M β-mercaptoethanol, pH 8.4) and used for two-photon excitation investigation. (a) Emission spectra of BSA in solution collected in the two-photon excitation range of 700 nm to 800 nm. Peak emission seen at around 460 nm, shifts slightly to longer emission wavelengths with the longer excitation wavelength. (b) Fluorescence lifetime histogram of BSA in solution at the excitation wavelength of 780 nm, collected in the blue (445/60 nm) channel. The average fluorescence lifetime seen at around 2.5 ns. The signal detected in the red (580/150 nm) channel was too weak for the fitting, in agreement with the emission spectra of BSA.

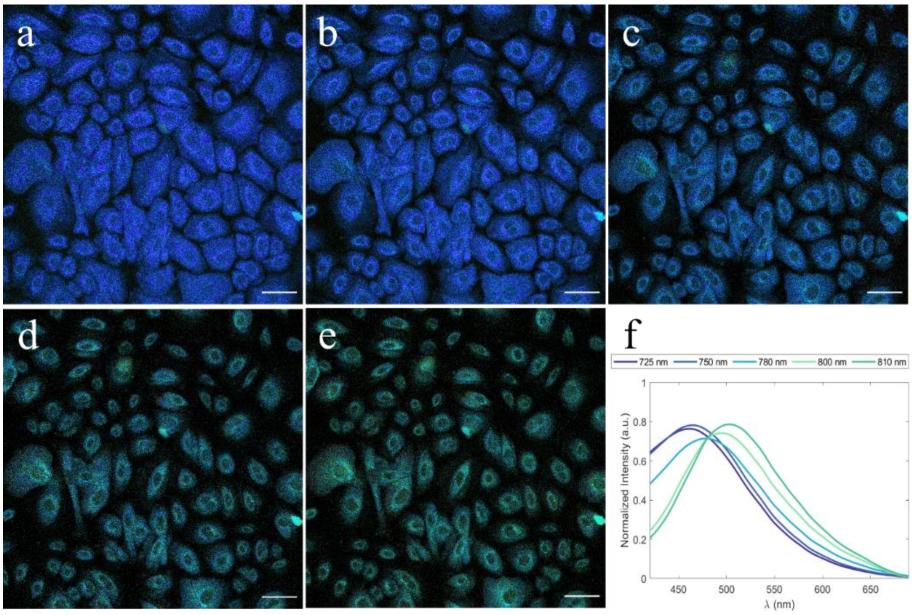

Fig. S5 Spectral MPM imaging of human keratinocytes. Color-coded lambda images of human keratinocytes collected at (A) 725 nm, (B) 750 nm, (C) 780 nm, (D) 800 nm, (E) 810 nm, and (F) corresponding emission spectra collected in the range of 421 nm to 693 nm. Scale bar: 50 μM.

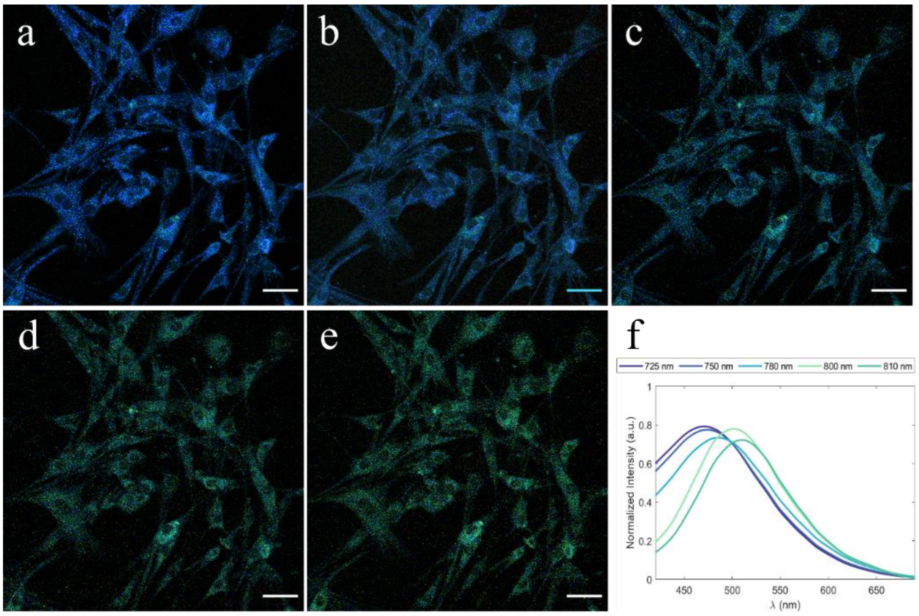

Fig. S6 Spectral MPM imaging of human fibroblasts. Color-coded lambda images of human fibroblasts collected at (A) 725 nm, (B) 750 nm, (C) 780 nm, (D) 800 nm, (E) 810 nm, and (F) corresponding emission spectra collected in the range of 421 nm to 693 nm. Scale bar: 50 µM.

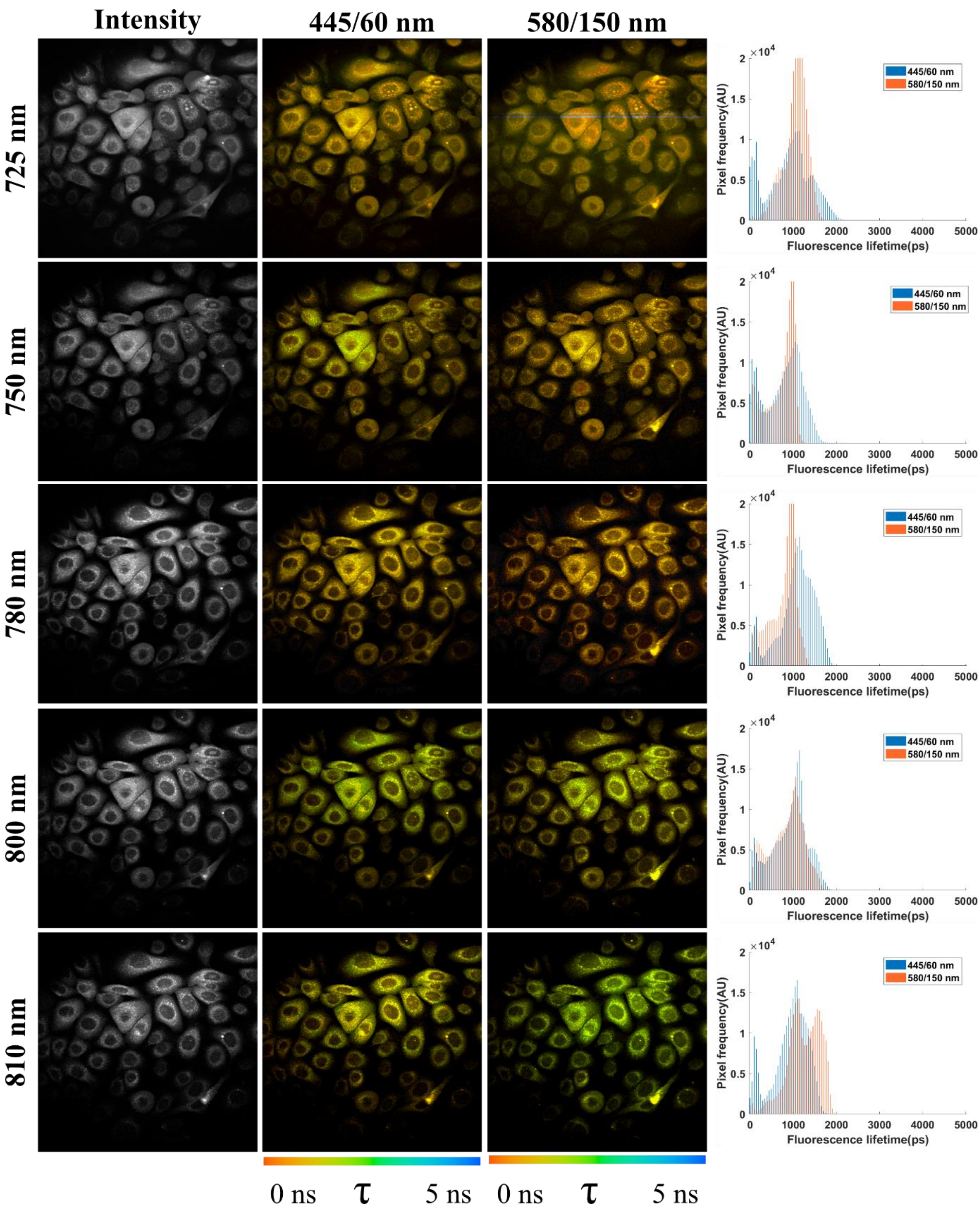

Fig. S7 MPM-FLIM imaging of human keratinocytes and fluorescence lifetime detection. Intensity and MPM-FLIM images of keratinocytes in the excitation range of 725 – 810 nm collected in the blue (445/60 nm) and red (580/150 nm) channel, and the corresponding lifetime histograms. The fluorescence lifetime range has been adjusted to 0 to 5 ns. Brightness and contrast in the images were adjusted for clarity. Field of view: ~ 350 x 350 µm^2. False-color scale fluorescence lifetime data, 256-time channels, ranging from 0 to 5 ns.

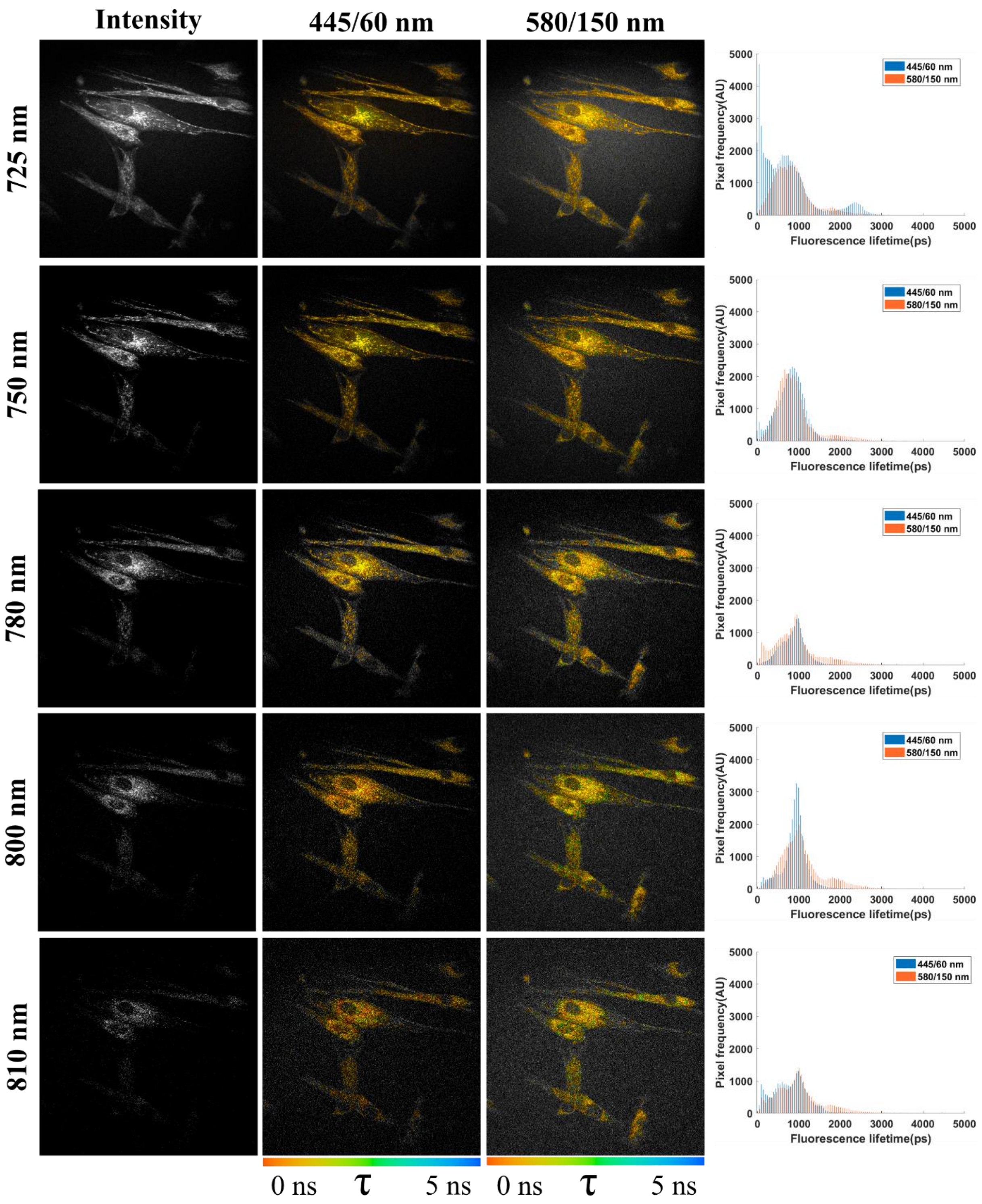

Fig. S8 MPM-FLIM imaging of human fibroblasts and fluorescence lifetime detection. Intensity and MPM-FLIM images of human fibroblasts in the excitation range of 725 – 810 nm collected in the blue (445/60 nm) and red (580/150 nm) channel, and the corresponding lifetime histograms. The fluorescence lifetime range has been adjusted to 0 to 5 ns. Brightness and contrast in the images were adjusted for clarity. Field of view: ~ 350 x 350 μm^2. False-color scale fluorescence lifetime data, 256-time channels, ranging from 0 to 5 ns.

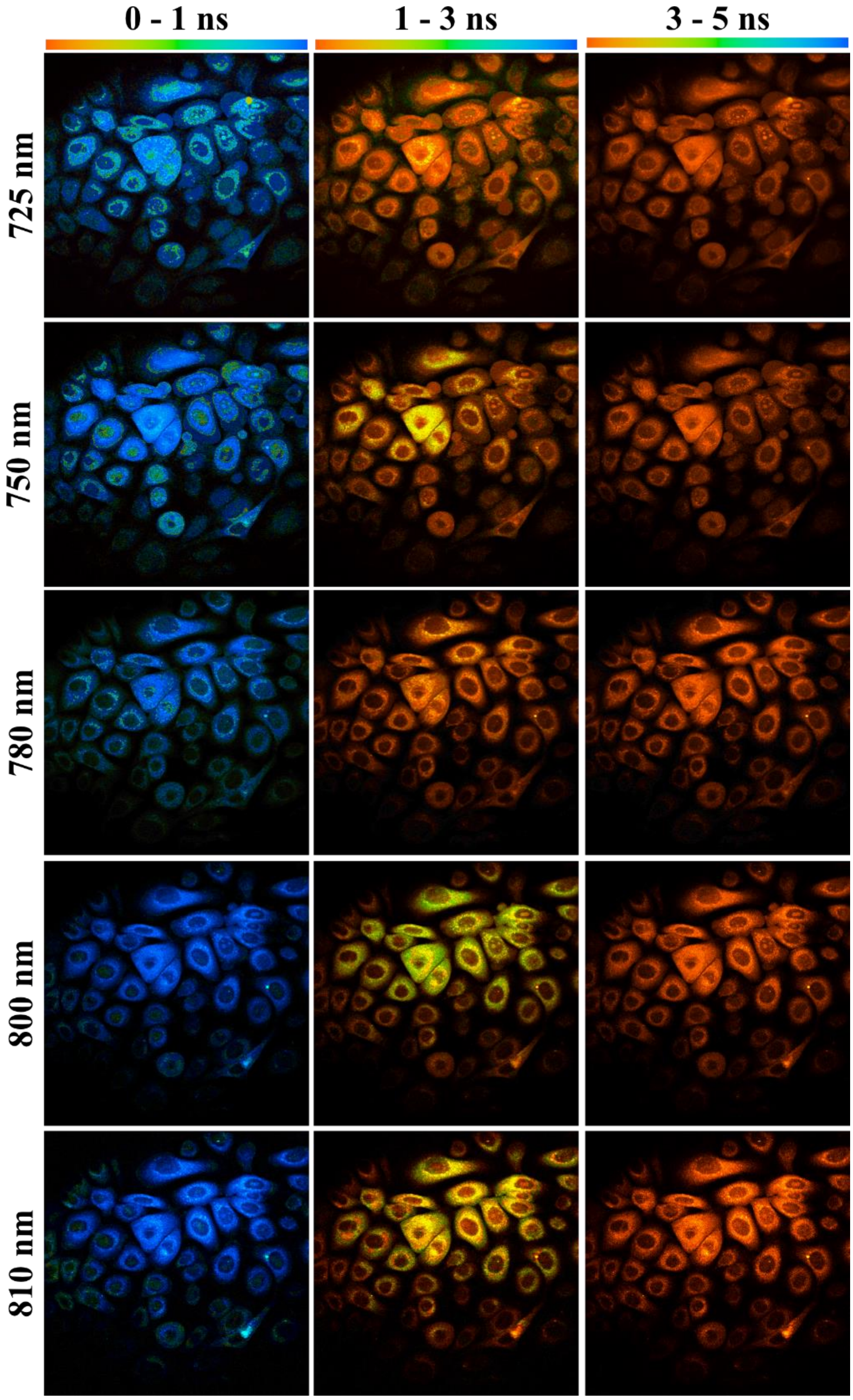

Fig. S9 Visual fluorescence lifetime separation of human keratinocytes in the blue channel. MPM-FLIM images of human keratinocytes in the excitation range of 725 – 810 nm collected in the blue channel. The fluorescence lifetime range has been adjusted to 0 to 1 ns, 1 to 3 ns, and 3 to 5 ns. Brightness and contrast in the images were adjusted for clarity. Field of view: ~ 350 x 350 μm^2. False-color scale fluorescence lifetime data, 256-time channels.

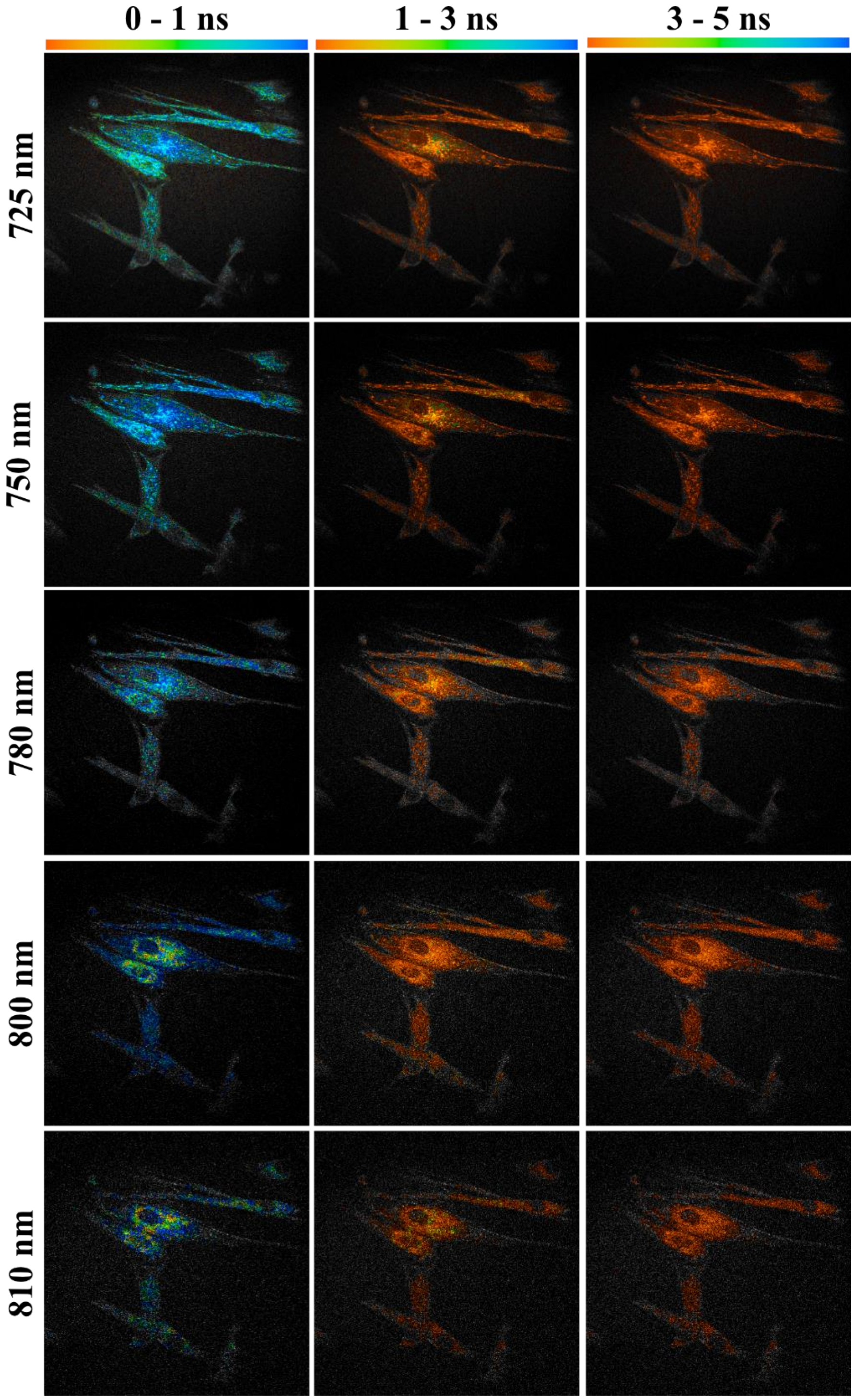

Fig. S10 Visual fluorescence lifetime separation of human fibroblasts in the blue channel. MPM-FLIM images of human fibroblasts in the excitation range of 725 – 810 nm collected in the blue channel. The fluorescence lifetime range has been adjusted to 0 to 1 ns, 1 to 3 ns, and 3 to 5 ns. Brightness and contrast in the images were adjusted for clarity. Field of view: ~ 350 x 350 μm^2. False-color scale fluorescence lifetime data, 256-time channels.

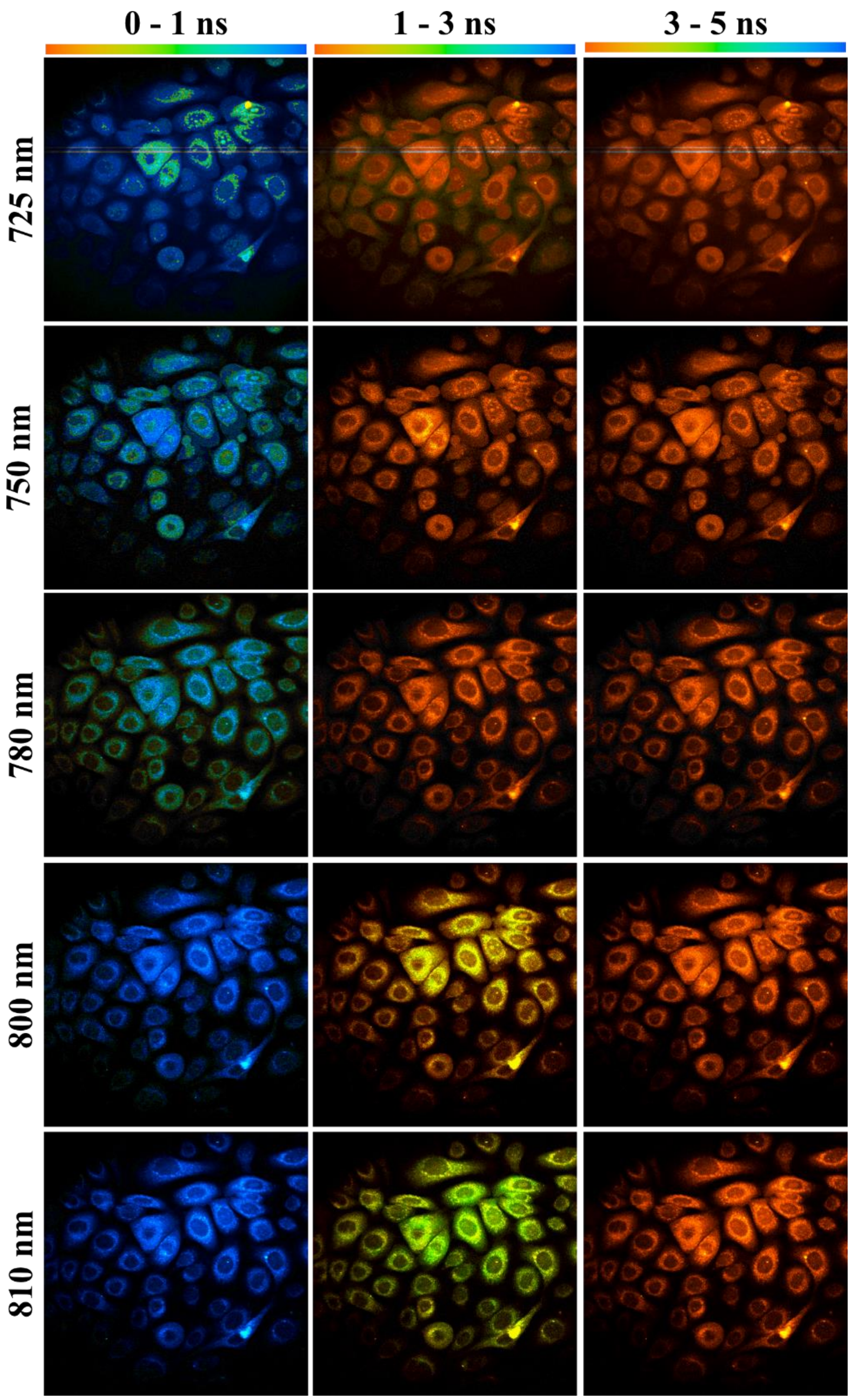

Fig. S11 Visual fluorescence lifetime separation of human keratinocytes in the red channel. MPM-FLIM images of keratinocytes in the excitation range of 725 – 810 nm collected in the red channel. The fluorescence lifetime range has been adjusted to 0 to 1 ns, 1 to 3 ns, and 3 to 5 ns. Brightness and contrast in the images were adjusted for clarity. Field of view: ~ 350 x 350 μm^2. False-color scale fluorescence lifetime data, 256-time channels.

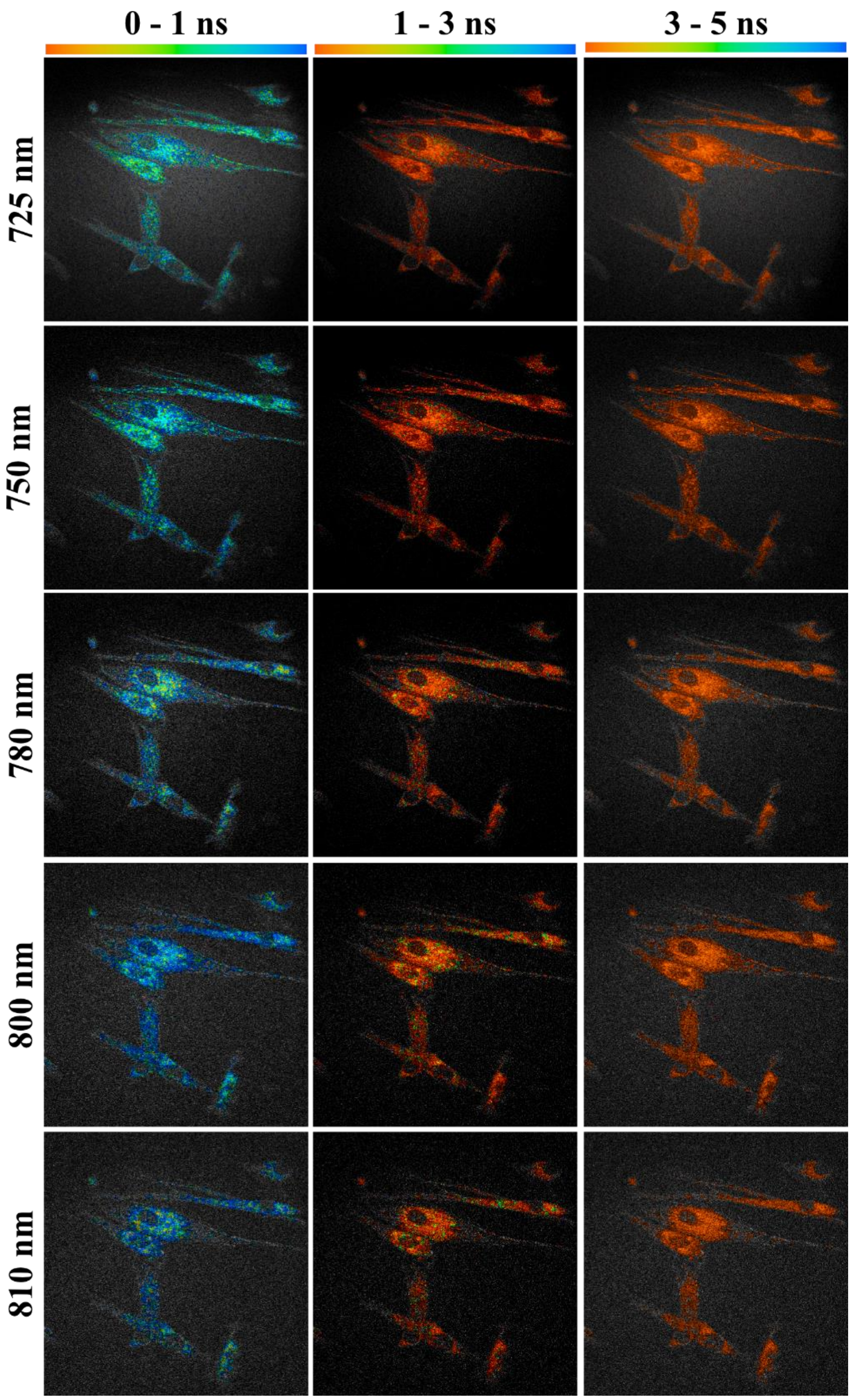

Fig. S12 Visual fluorescence lifetime separation of human fibroblasts in the red channel. MPM-FLIM images of fibroblasts in the excitation range of 725 – 810 nm collected in the red channel. The fluorescence lifetime range has been adjusted to 0 to 1 ns, 1 to 3 ns, and 3 to 5 ns. Brightness and contrast in the images were adjusted for clarity. Field of view: ~ 350 x 350 µm^2. False-color scale fluorescence lifetime data, 256-time channels.

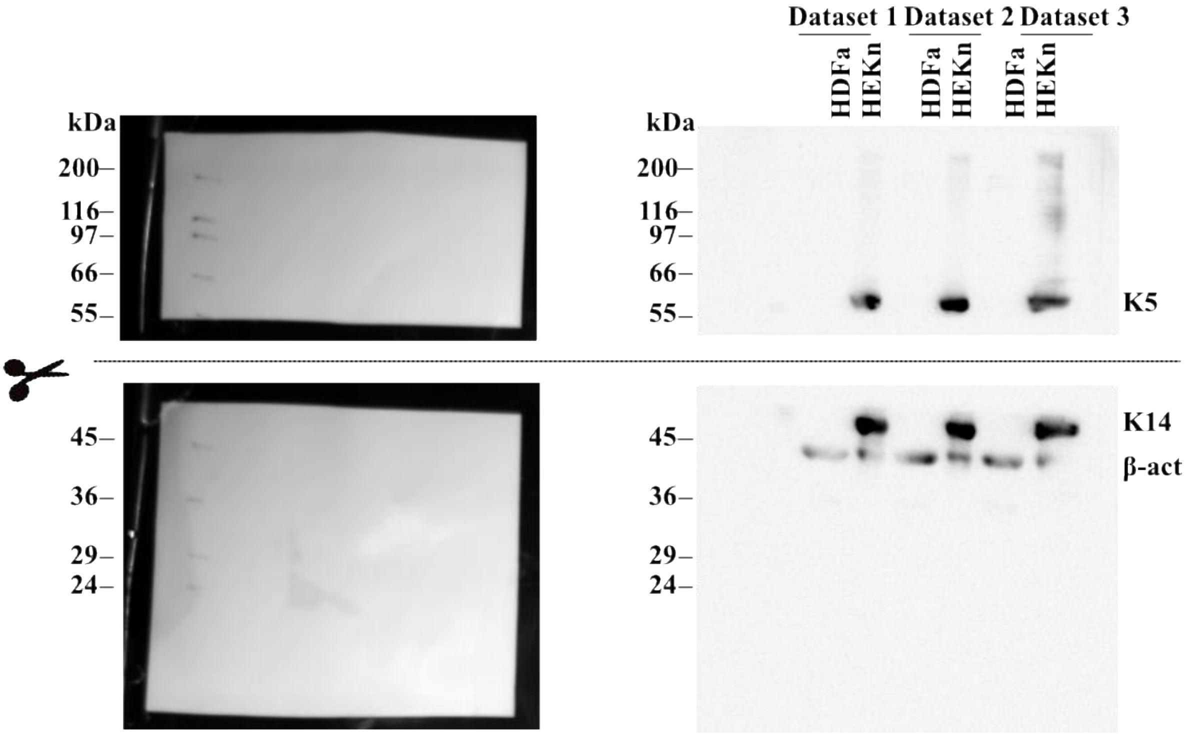

Fig. S13 Western blot analysis of human keratinocytes and human fibroblasts. *Left panel:* After blotting, the membrane was cut in half at the marker band of 55 kDa, according to the protein marker, which is marked in pencil following staining with Ponceau S. *Right panel:* The membrane containing proteins larger in size than 55 kDa was probed with an anti-cytokeratin 5 antibody, followed by an incubation with a secondary antibody. The membrane containing proteins smaller in size than 55 kDa was probed simultaneously with anti-cytokeratin 14 and anti-β-actin antibody, followed by simultaneous incubation with the secondary antibodies. Three datasets were probed simultaneously, each of them containing protein extracts from human fibroblasts and human keratinocytes. The membranes were imaged with the same exposure time (2 min.).

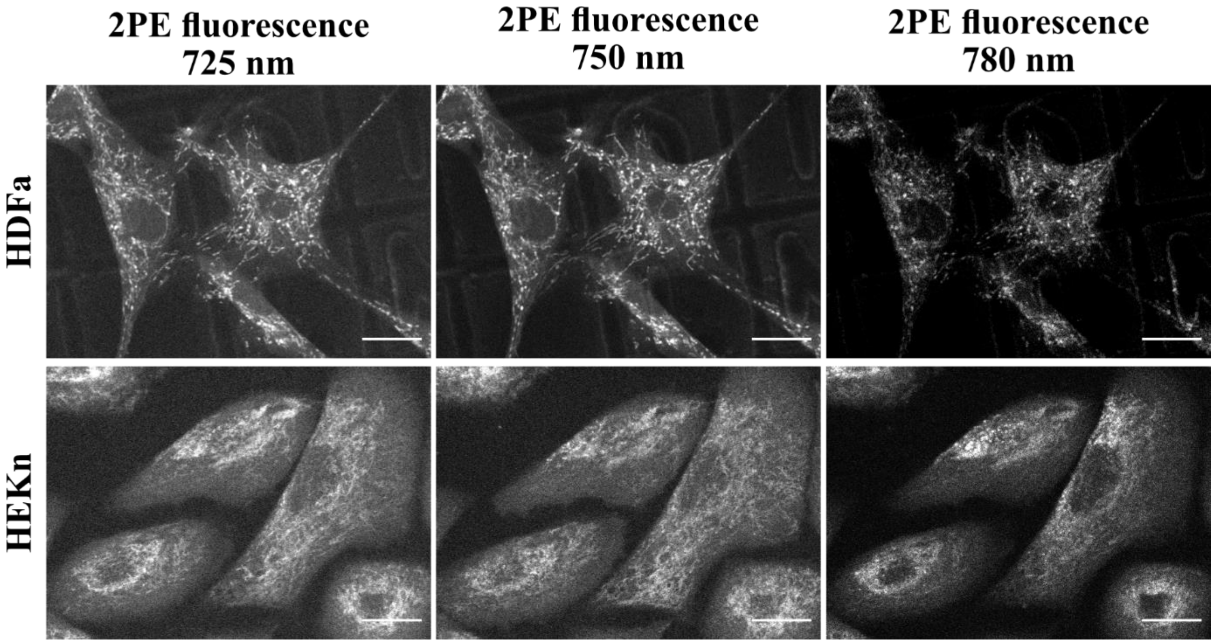

Fig. S14 2PE autofluorescence of human fibroblasts and human keratinocytes in the range 725 – 780 nm. Autofluorescence MPM of cells collected with a GaAsP detector in the excitation range of 725 – 780 nm. Brightness and contrast in the images were adjusted for clarity. Scale bar 10 µm.

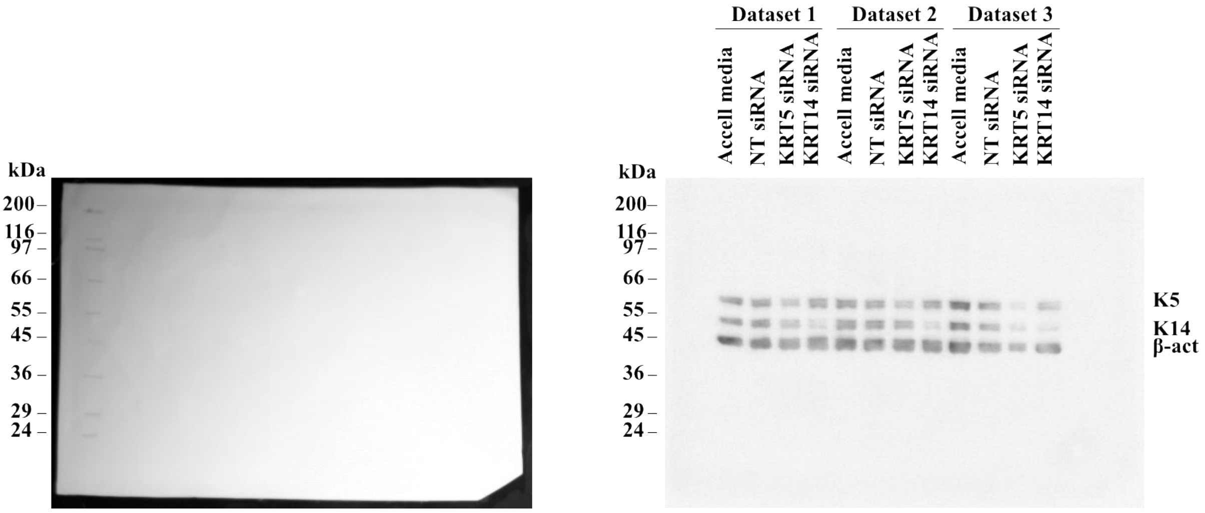

Fig. S15 Western blot analysis of siRNA silenced human keratinocytes. *Left panel:* The size bands were marked according to the protein marker, which is marked in pencil following staining with Ponceau S. *Right panel:* Three datasets were analyzed simultaneously, each of them containing (from left to right): human keratinocytes grown in Accell® siRNA delivery growth medium, human keratinocytes incubated with non-targeting siRNA (NT siRNA), human keratinocytes incubated with KRT5 siRNA, and human keratinocytes incubated with KRT14 siRNA. NT siRNA samples were used as a control. The membrane was probed simultaneously with an anti-cytokeratin 5, anti-cytokeratin 14 and anti-β-actin antibodies, followed by simultaneous incubation with the secondary antibodies.

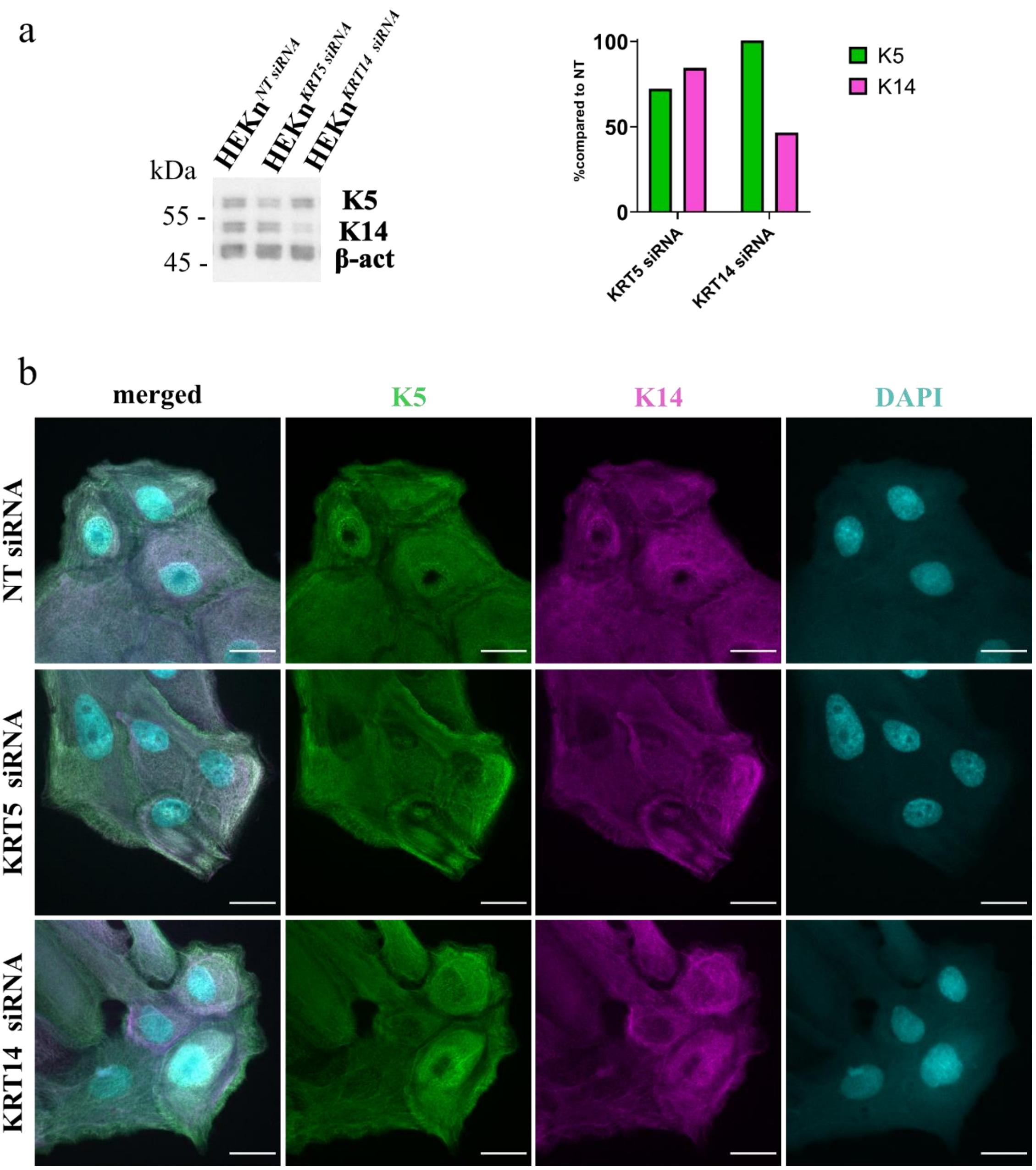

Fig. S16 Characterization of keratin levels and keratin intermediate filaments in siRNA transfected keratinocytes (dataset 2). (a) Depletion of keratin 5 and keratin 14 in keratinocytes analyzed by Western blotting where non-targeting siRNA samples are used as a control. The primary antibodies were incubated simultaneously, as were the secondary antibodies. Protein loadings were standardized for total protein content based on Coomassie blue stained gels and densitometric analysis. An antibody to β actin was used on the same membrane to demonstrate even loading. (b) Confocal images of HEKn$^{NT\ siRNA}$, HEKn$^{KRT5\ siRNA}$ and HEKn$^{KRT14\ siRNA}$ stained for keratin 5, keratin 14 and DAPI. Images were merged to visualize keratin 5 and keratin 14 co-localization. Scale bar: 10 μm.

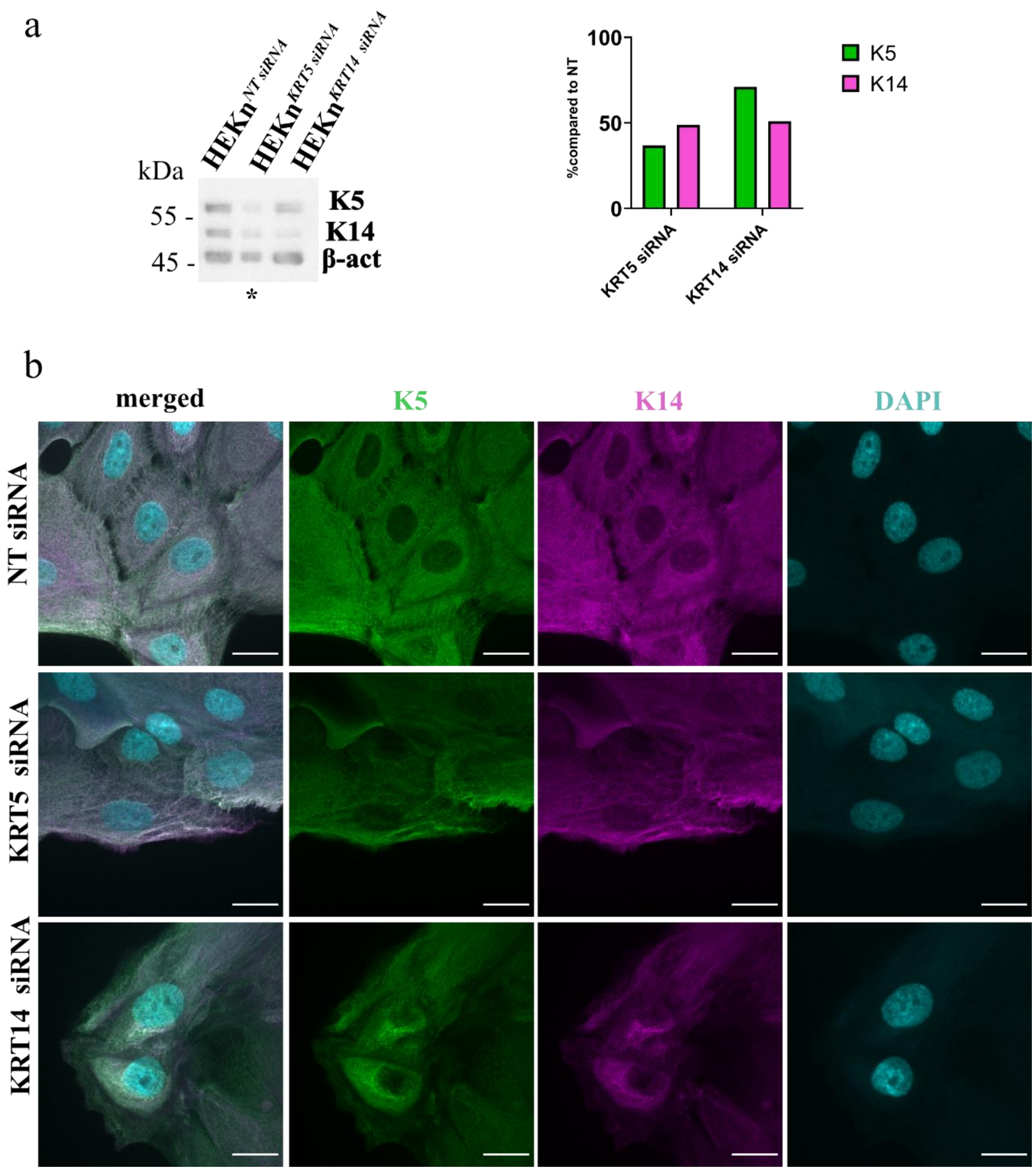

Fig. S17 Characterization of keratin levels and keratin intermediate filaments in siRNA transfected keratinocytes (dataset 3). (a) Depletion of keratin 5 and keratin 14 in keratinocytes analyzed by Western blotting where non-targeting siRNA samples are used as a control. The primary antibodies were incubated simultaneously, as were the secondary antibodies. Protein loadings were standardized for total protein content based on Coomassie blue stained gels and densitometric analysis. An antibody to β actin was used on the same membrane to demonstrate even loading. Note: the protein content of $HEKn^{KRT5\ siRNA}$ was lower than the other samples; however, the levels of keratin proteins were significantly lower than in $HEKn^{NT\ siRNA}$. (b) Confocal images of $HEKn^{NT\ siRNA}$, $HEKn^{KRT5\ siRNA}$ and $HEKn^{KRT14\ siRNA}$ stained for keratin 5, keratin 14 and DAPI. Images were merged to visualize keratin 5 and keratin 14 co-localization. Scale bar: 10 µm.

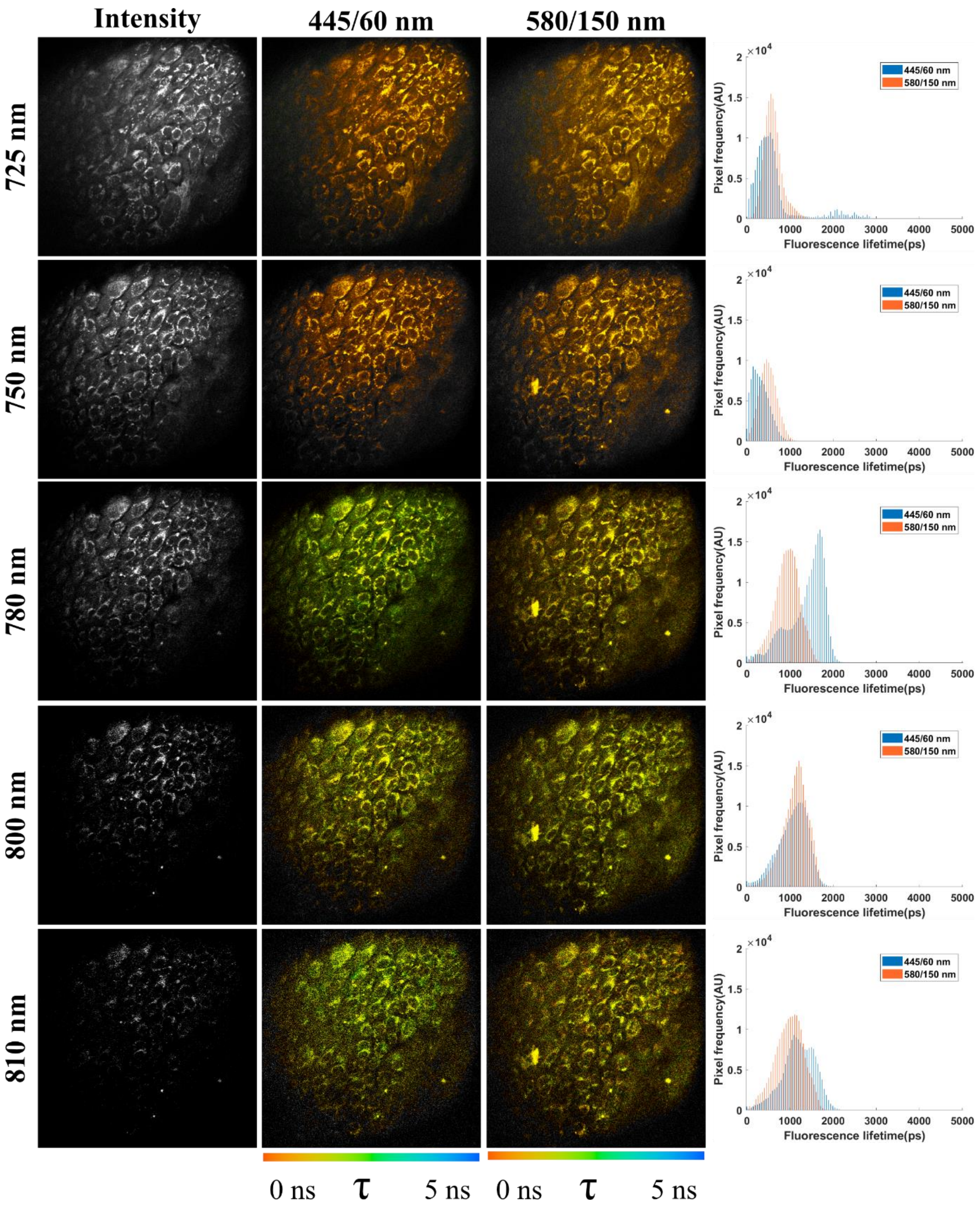

Fig. S18 MPM-FLIM imaging of human keratinocytes transfected with non-targeting siRNA. Intensity and MPM-FLIM images of keratinocytes transfected with a non-targeting siRNA, in the excitation range of 725 – 810 nm collected in the blue (445/60 nm) and red (580/150 nm) channel, and the corresponding lifetime histograms. The fluorescence lifetime range has been adjusted to 0 to 5 ns. Brightness and contrast in the images were adjusted

for clarity. Field of view: ~ 350 x 350 µm^2. False-color scale fluorescence lifetime data, 256-time channels, ranging from 0 to 5 ns.

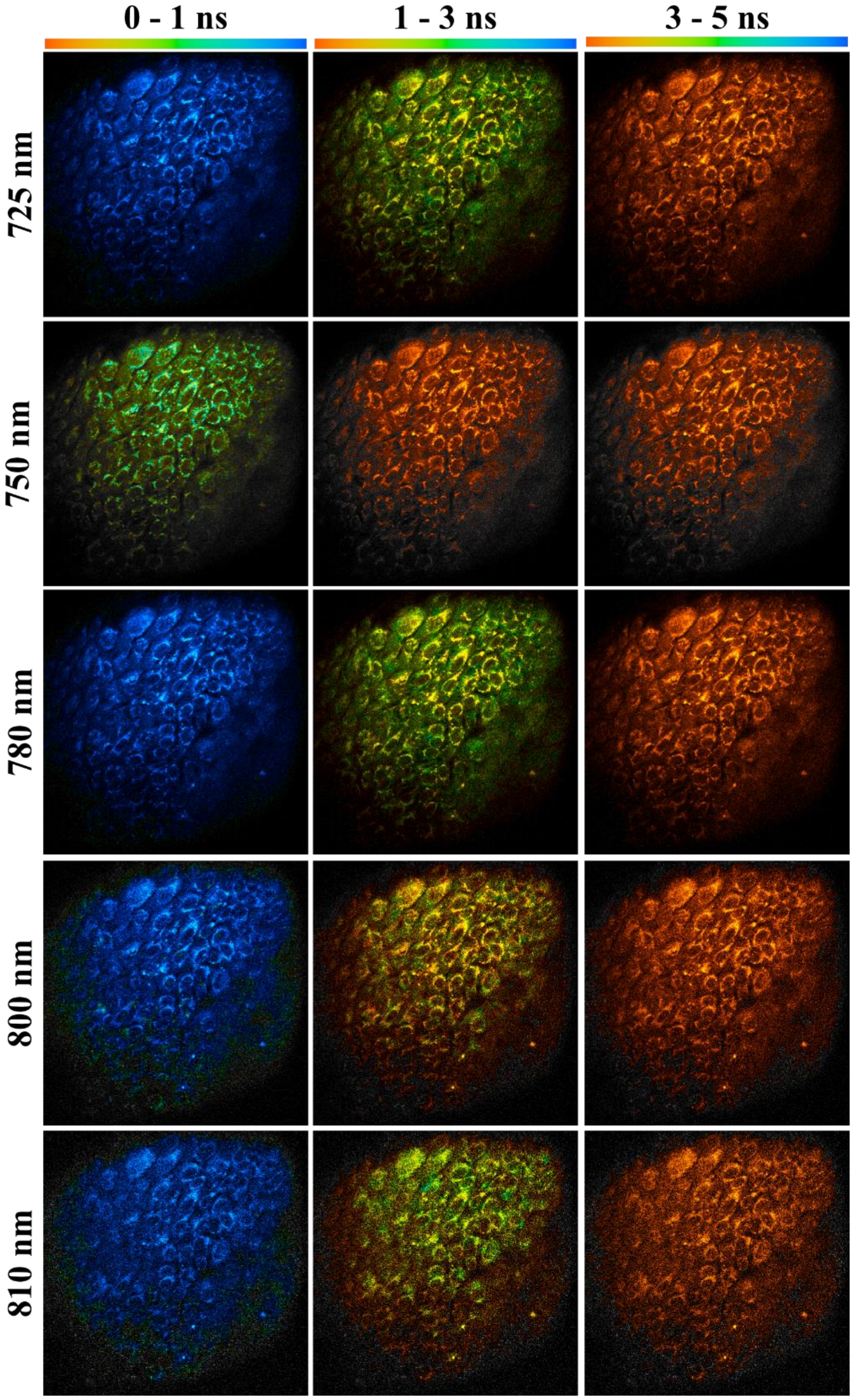

Fig. S19 Visual fluorescence lifetime separation in the blue channel of human keratinocytes transfected with non-targeting siRNA. MPM-FLIM images of keratinocytes in the excitation range of 725 – 810 nm collected in the red channel. The fluorescence lifetime range has been adjusted to 0 to 1 ns, 1 to 3 ns, and 3 to 5 ns. Brightness and contrast in the images were adjusted for clarity. Field of view: ~ 350 x 350 µm^2. False-color scale fluorescence lifetime data, 256-time channels.

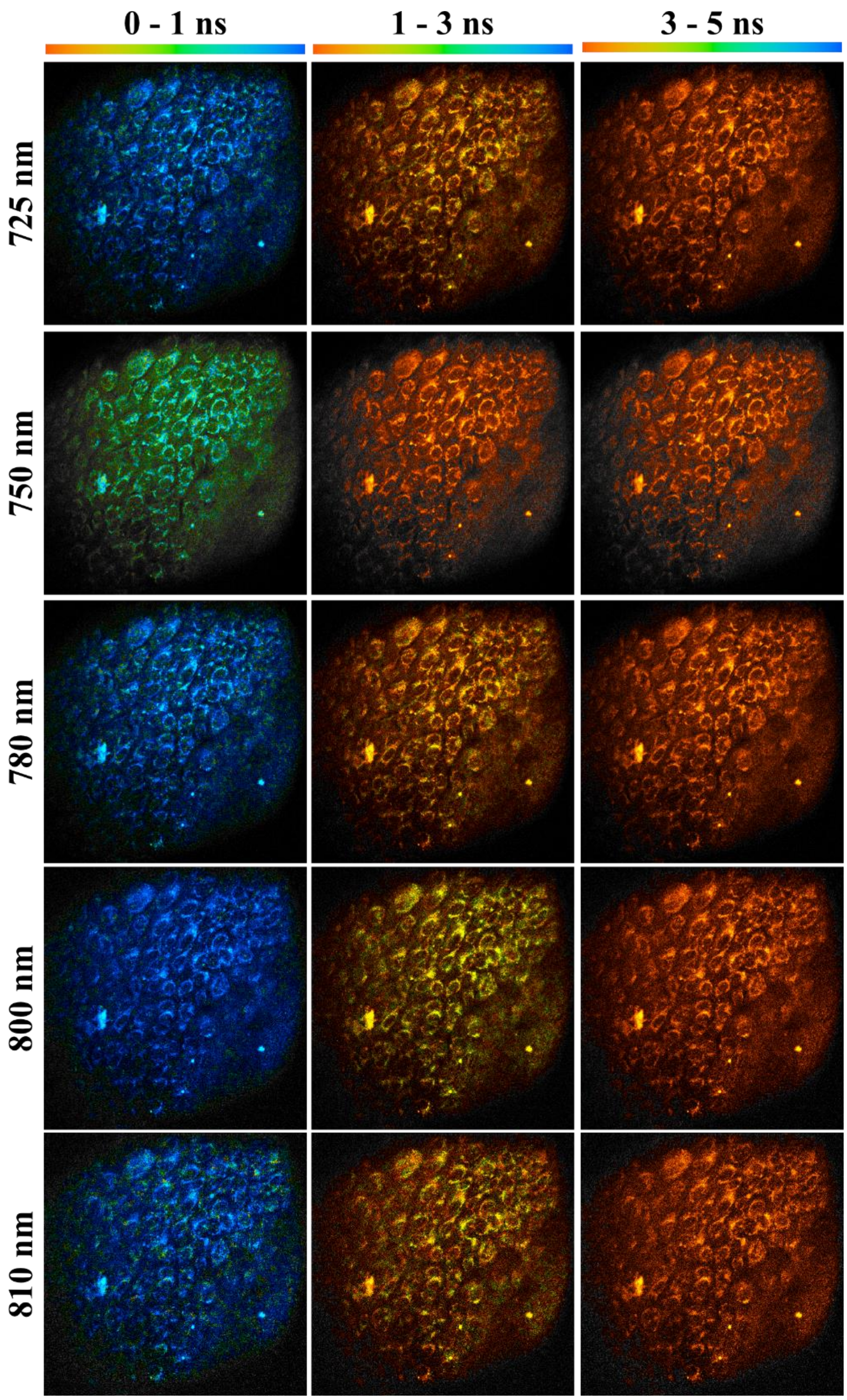

Fig. S20 Visual fluorescence lifetime separation in the red channel of human keratinocytes transfected with non-targeting siRNA. MPM-FLIM images of keratinocytes in the excitation range of 725 – 810 nm collected in the red channel. The fluorescence lifetime range has been adjusted to 0 to 1 ns, 1 to 3 ns, and 3 to 5 ns. Brightness and contrast in the images were adjusted for clarity. Field of view: ~ 350 x 350 µm^2. False-color scale fluorescence lifetime data, 256-time channels.

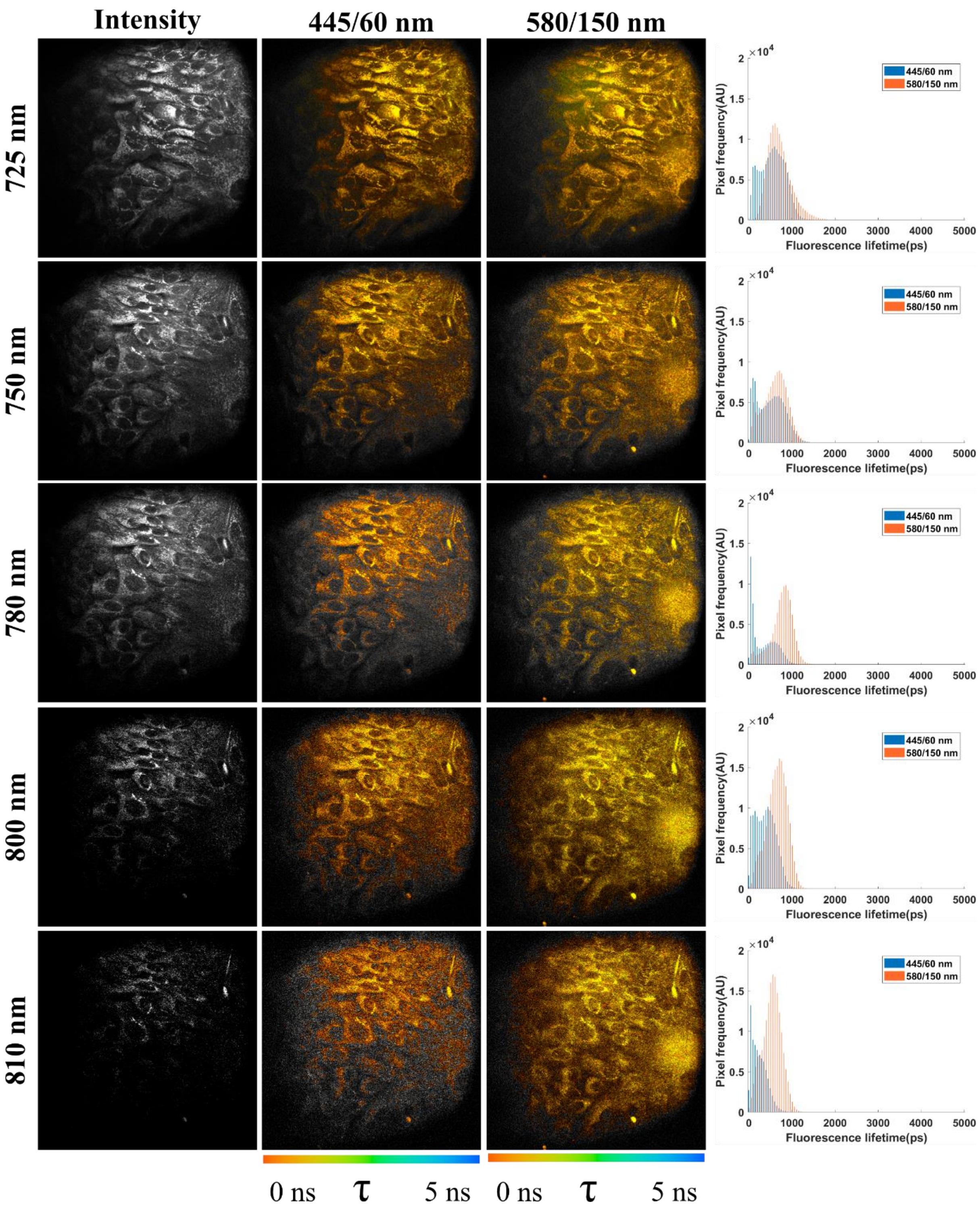

Fig. S21 MPM-FLIM imaging of human keratinocytes transfected with KRT5 siRNA. Intensity and MPM-FLIM images of keratinocytes transfected with KRT5 siRNA, in the excitation range of 725 – 810 nm collected in the blue (445/60 nm) and red (580/150 nm) channel, and the corresponding lifetime histograms. The fluorescence lifetime range has been adjusted to 0 to 5 ns. Brightness and contrast in the images were adjusted for clarity. Field of view: ~ 350 x 350 μm^2. False-color scale fluorescence lifetime data, 256-time channels, ranging from 0 to 5 ns.

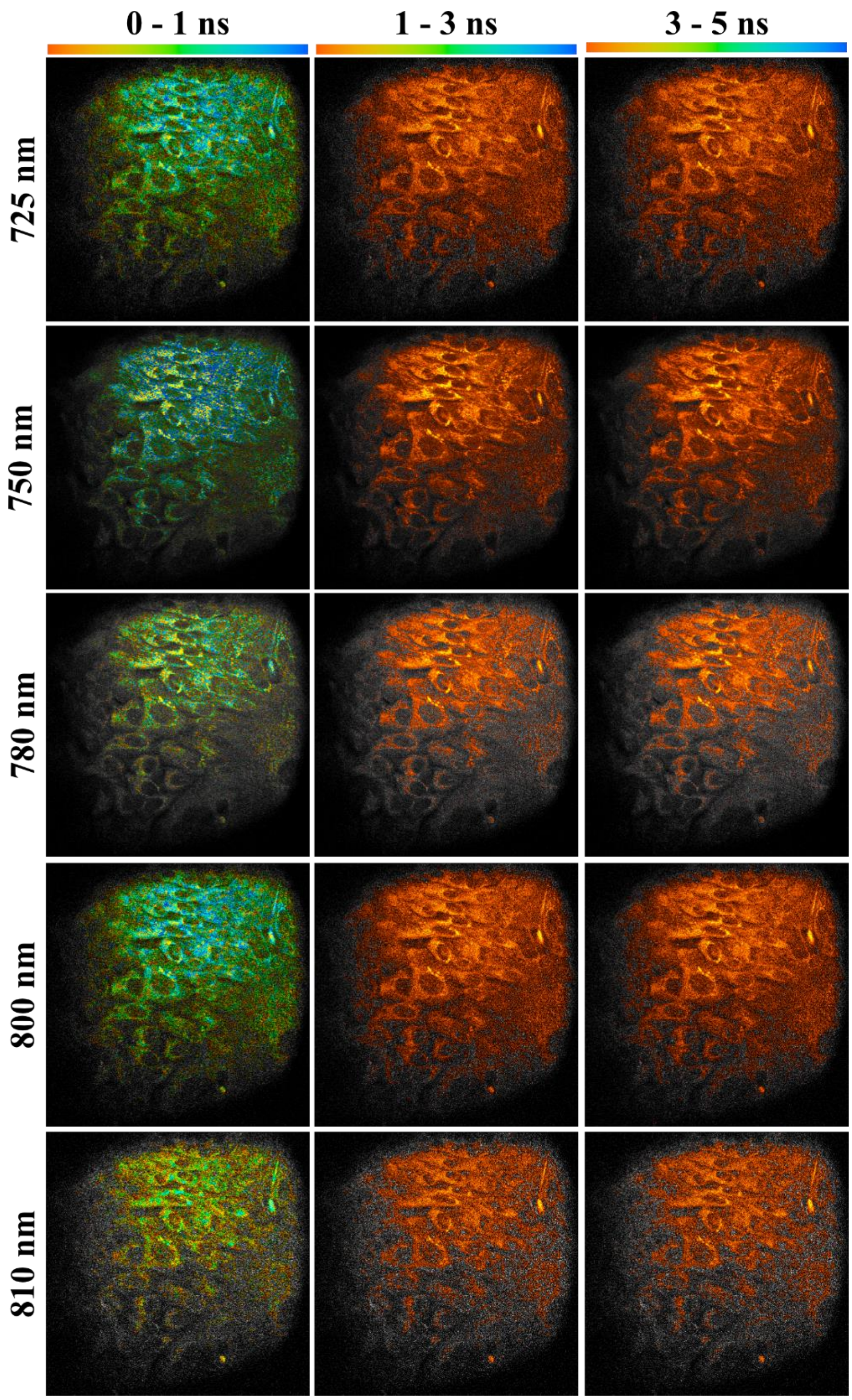

Fig. S22 Visual fluorescence lifetime separation in the blue channel of human keratinocytes transfected with KRT5 siRNA. MPM-FLIM images of keratinocytes in the excitation range of 725 – 810 nm collected in the blue channel. The fluorescence lifetime range has been adjusted to 0 to 1 ns, 1 to 3 ns, and 3 to 5 ns. Brightness and contrast in the images were adjusted for clarity. Field of view: ~ 350 x 350 μm^2. False-color scale fluorescence lifetime data, 256-time channels.

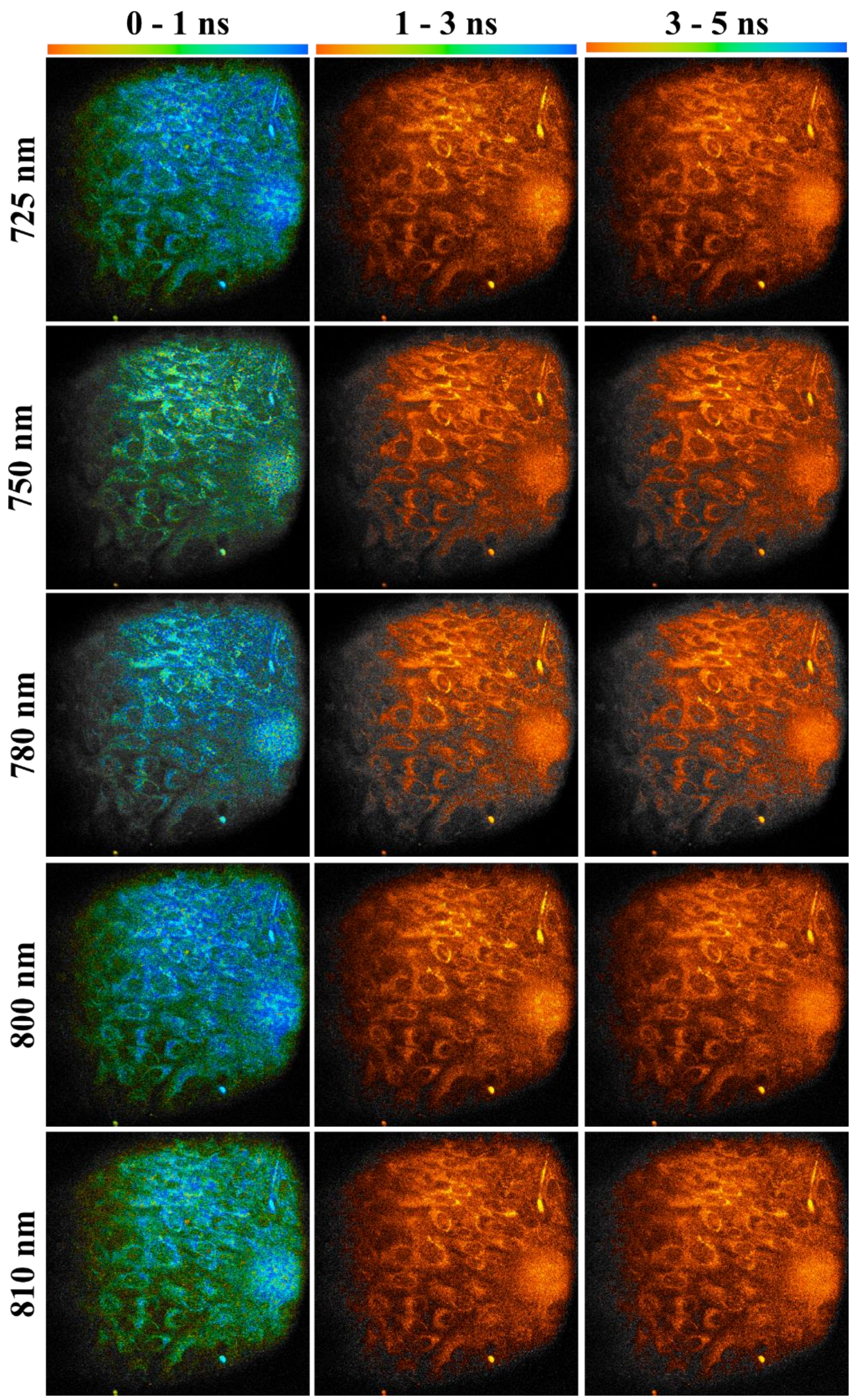

Fig. S23 Visual fluorescence lifetime separation in the red channel of human keratinocytes transfected with KRT5 siRNA. MPM-FLIM images of keratinocytes in the excitation range of 725 – 810 nm collected in the red channel. The fluorescence lifetime range has been adjusted to 0 to 1 ns, 1 to 3 ns, and 3 to 5 ns. Brightness and contrast in the images were adjusted for clarity. Field of view: ~ 350 x 350 μm^2. False-color scale fluorescence lifetime data, 256-time channels.

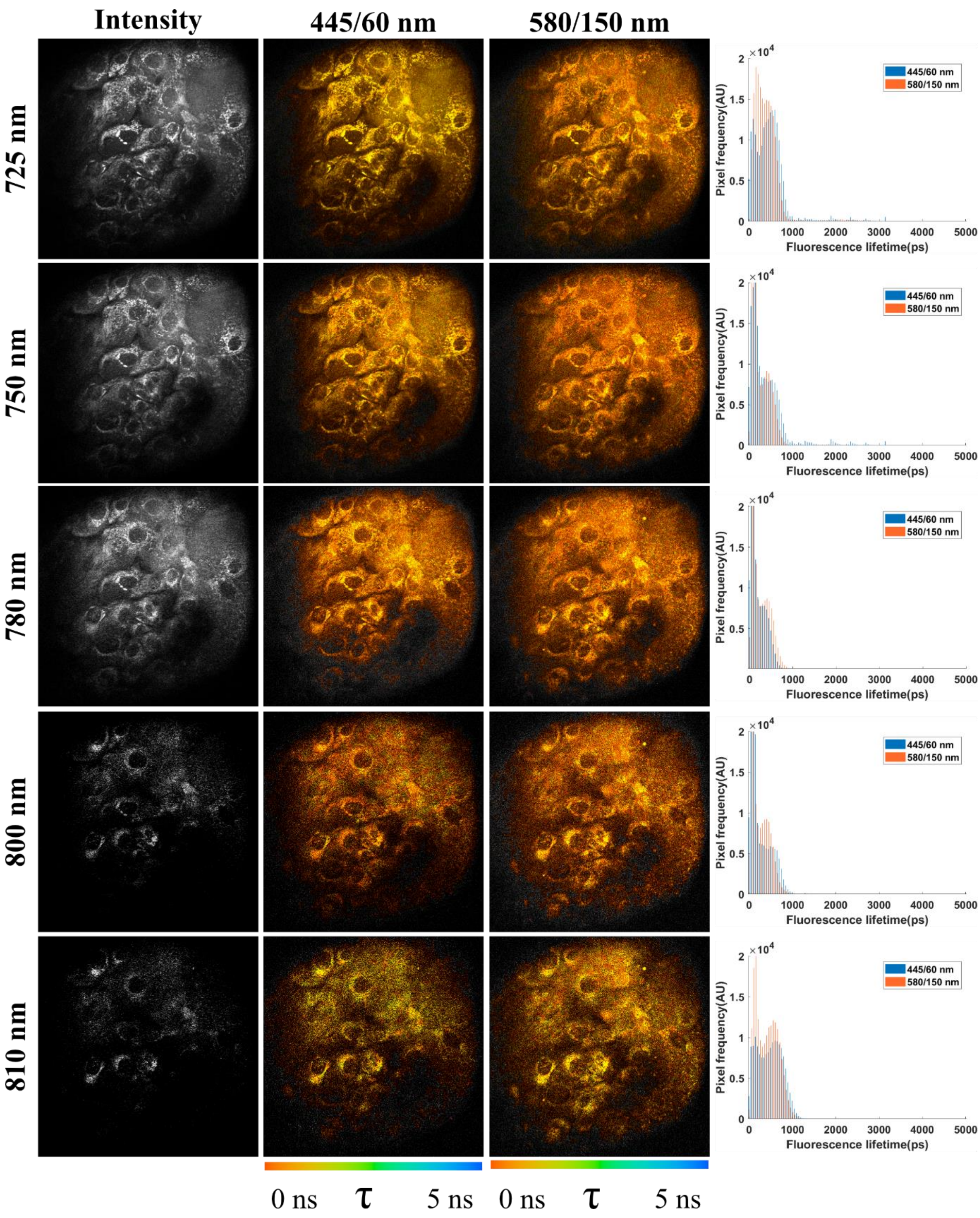

Fig. S24 MPM-FLIM imaging of human keratinocytes transfected with KRT14 siRNA. Intensity and MPM-FLIM images of keratinocytes transfected with KRT14 siRNA, in the excitation range of 725 – 810 nm collected in the blue (445/60 nm) and red (580/150 nm) channel, and the corresponding lifetime histograms. The fluorescence lifetime range has been adjusted to 0 to 5 ns. Brightness and contrast in the images were adjusted for clarity. Field of view: ~ 350 x 350 μm^2. False-color scale fluorescence lifetime data, 256-time channels, ranging from 0 to 5 ns.

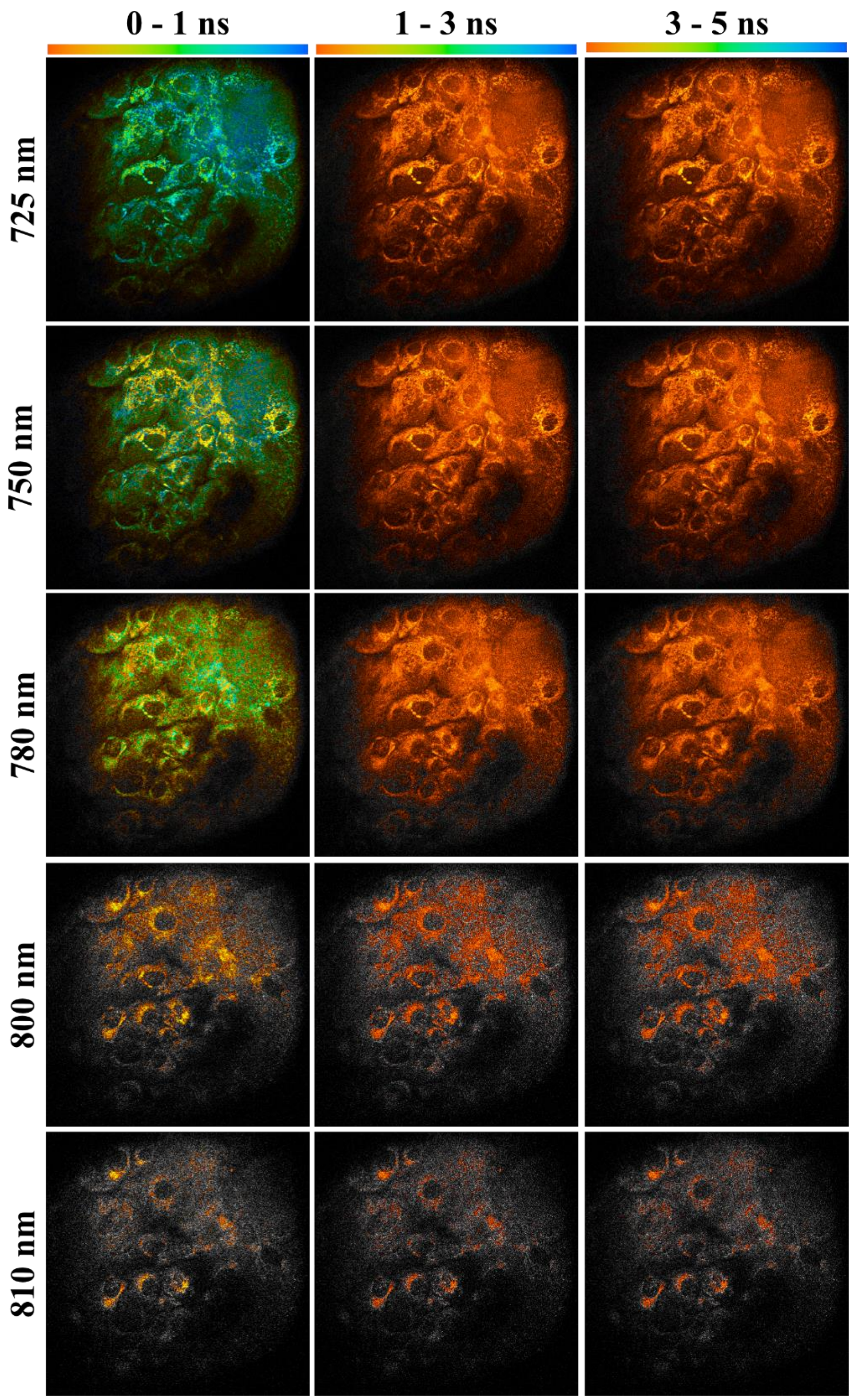

Fig. S25 Visual fluorescence lifetime separation in the blue channel of human keratinocytes transfected with KRT14 siRNA. MPM-FLIM images of keratinocytes in the excitation range of 725 – 810 nm collected in the blue channel. The fluorescence lifetime range has been adjusted to 0 to 1 ns, 1 to 3 ns, and 3 to 5 ns. Brightness and contrast in the images were adjusted for clarity. Field of view: ~ 350 x 350 μm^2. False-color scale fluorescence lifetime data, 256-time channels.

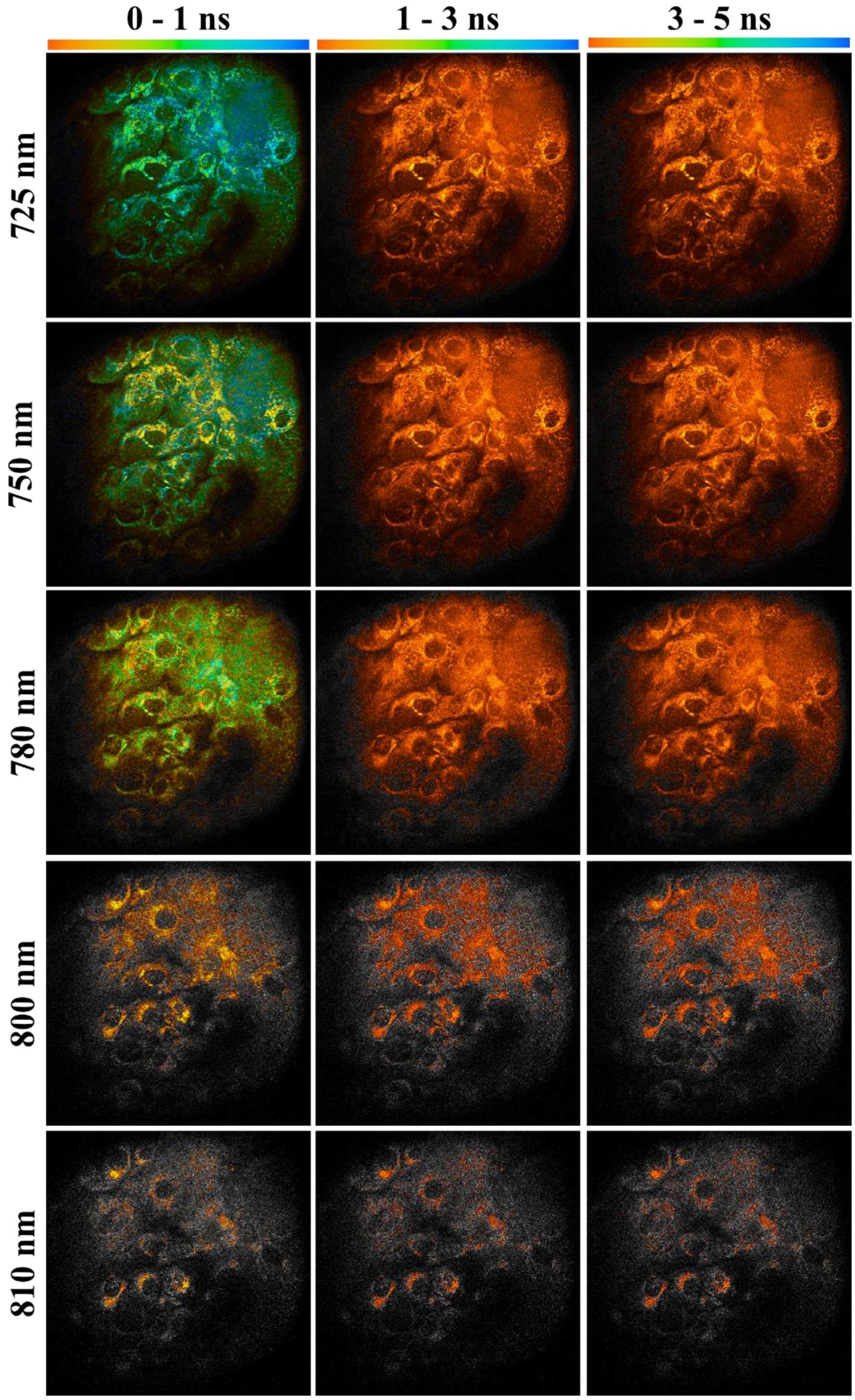

Fig. S26 Visual fluorescence lifetime separation in the red channel of human keratinocytes transfected with KRT14 siRNA. MPM-FLIM images of keratinocytes in the excitation range of 725 – 810 nm collected in the red channel. The fluorescence lifetime range has been adjusted to 0 to 1 ns, 1 to 3 ns, and 3 to 5 ns. Brightness and contrast in the images were adjusted for clarity. Field of view: ~ 350 x 350 μm^2. False-color scale fluorescence lifetime data, 256-time channels.

a

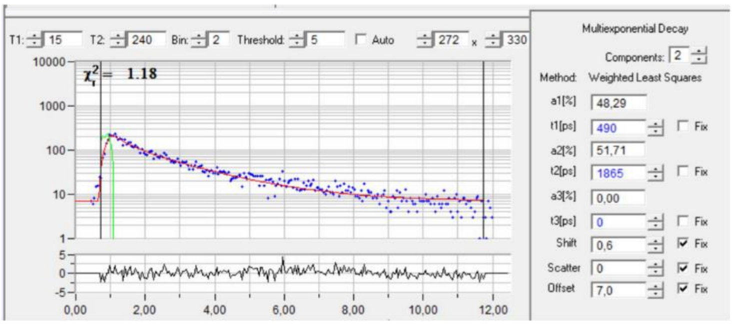

b

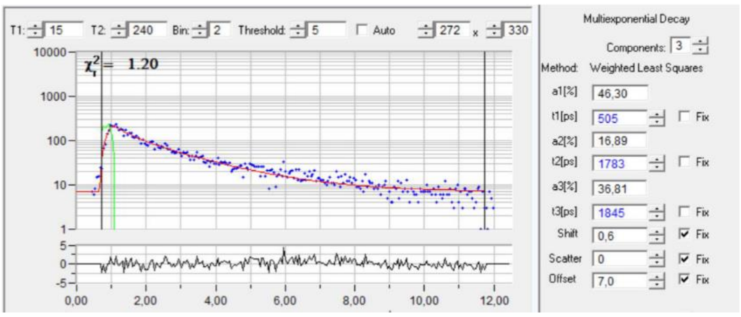

Fig. S27 Fitting comparison. Fluorescence decay curve showing (a) biexponential and (b) triexponential decay model. Blue: photons collected, green: instrument response function and red: fitted decay curve.
